# Native Starch Granules from Unconventional Peruvian Sources: Banana, Mango and Taro

**DOI:** 10.3390/polym18182252

**Published:** 2026-09-15

**Authors:** Paola Ortiz-Escobar, Hilka Mariela Carrion-Sanchez, Lucero Quispe Chambilla, Carmen Velezmoro-Sanchez, Ivo Mottin Demiate, Luiz Gustavo Lacerda, Sylvia Carolina Alcazar-Alay, Augusto Pumacahua-Ramos

**Affiliations:** 1 Department of Food Engineering, Universidad Nacional Intercultural de Quillabamba (UNIQ), Quillabamba 08741, Peru; hilka.carrion@uniq.edu.pe (H.M.C.-S.); lucero.quispe@uniq.edu.pe (L.Q.C.); augusto.pumacahua@uniq.edu.pe (A.P.-R.); 2 Food Science and Technology Graduate Program, Universidade Estadual de Ponta Grossa (UEPG), Ponta Grossa 84030-900, Brazil; demiate@uepg.br (I.M.D.); luizgustavo75@gmail.com (L.G.L.); 3 Departamento de Ingeniería de alimentos y Productos Agropecuarios, Universidad Nacional Agraria La Molina (UNALM), Lima 12056, Peru; cevs@lamolina.edu.pe

**Keywords:** starch, structural properties, rheological properties, thermal properties, crystallinity, stability

## Abstract

The transition toward sustainable food systems has driven the use of agricultural materials and agroindustrial byproducts as alternative sources of functional ingredients. In this study, starches were isolated from Bellaco plantains, Criollo mango seeds, and Antiquorum taro using aqueous extraction, and their physicochemical, morphological, structural, rheological, and thermal properties were characterized. The amylose content ranged from 16.83% to 25.92%, values comparable to those of conventional starch sources. Taro starch exhibited the highest brightness (98.30) and whiteness index (96.56). Plantain starch exhibited larger granules, ranging in shape from oval to ellipsoidal (24.37 μm), followed by mango seed starch (15.21 μm), while taro starch had smaller, irregular, polygonal granules (6.64 μm). The diffraction patterns were of types A and C, and the Fourier-transform infrared (FTIR) spectra confirmed bands characteristic of starchy materials. Swelling, solubility, and water absorption increased with temperature, with distinct responses at 90 °C. Plantain starch exhibited the highest peak viscosity (7003.67 cP) and setback viscosity (3789.67 cP). All starches exhibited elastic behavior, with the storage modulus (G′) exceeding the loss modulus (G″), gelatinization temperatures ranging from 70.03 to 72.86 °C, different energy requirements for crystalline disruption, and maximum thermal degradation temperatures ranging from 315.58 to 319.27 °C. These results demonstrate the potential of these starches as alternative functional ingredients.

## 1. Introduction

Constant pressure on natural resources, driven by population growth and agroindustrial intensification, has placed the agri-food system in a critical situation in terms of sustainability, availability of raw materials, and food losses [1,2]. In response, there has been growing interest in the valorization of food resources and byproducts as a potential source of high-value-added compounds to meet the growing demand for food [3,4].

Among naturally occurring polymers, starch is a key polysaccharide that stands out for its widespread availability in production systems, low cost of production, and suitability for technological integration, as well as its biodegradable, non-toxic nature and high compatibility with biological matrices, which has led to its increasing use in various food and industrial sectors [5,6]. Based on its macromolecular fraction, starch is classified as an energy-storage polysaccharide [7], consisting of α (1 → 4) and α (1 → 6) glycosidic bonds, in both linear (amylose) and branched (amylopectin) [8,9,10]. The proportion, organization, and interactions of these components are decisive factors governing the hydration, gelatinization, and viscoelastic behavior of starch-based systems [11,12].

Conventionally, starch production relies on a limited number of sources, such as corn, cassava, wheat, and potatoes, reaching a production level of up to 97.7 Mt in 2020 [13]. This dependence is associated with extensive monocultures, limited diversification, and intensive use of resources [14,15]. In this context, various studies have focused on exploring non-traditional sources of starch derived from little-studied plant resources, as well as agroindustrial waste generated throughout the production chain [16,17,18].

Within the framework of agricultural diversification and valorization, the eastern slope of the Peruvian Andes is a strategic area for the conservation and utilization of plant species and varieties adapted to a wide range of environmental and altitudinal conditions [19]. Among these resources, the ‘Bellaco’ plantain, the ‘Criollo’ mango, and taro (*Colocasia esculenta* var. *antiquorum*) stand out for their presence in traditional agricultural systems and for their potential as unconventional sources of starch, whose structural and functional properties remain relatively unexplored.

The banana (*Musa paradisiaca*) is one of the world’s most dynamic agricultural products; its production exceeded 124 million metric tons in 2021, and exports accounted for 19.67% of the total [20]. In Peru, banana production is highly productive and accounts for 3% of the international market [21,22]. Among locally cultivated varieties, the “Bellaco” banana (genomic group AAB) stands out for its importance in production and consumption. However, its climacteric nature and high perishability contribute to losses of between 10% and 15% throughout the production chain [23]. When unripe, its pulp contains between 70% and 80% starch on a dry-matter basis [24], highlighting its potential as a source of starch for food and industrial applications.

Likewise, the mango (*Mangifera indica*) is a tropical fruit of commercial importance in more than 100 countries [25], and Peru ranks third among global exporters [26]. In addition to widely marketed varieties, the “Criollo” variety is a mango population intended primarily for local consumption. However, despite the fruit’s availability, there is a scarcity of studies focused on this variety, limiting a comprehensive understanding of its functional qualities; furthermore, its postharvest handling faces phytosanitary restrictions associated with its high susceptibility to pests such as the fruit fly. As a result, its processing and consumption generate considerable amounts of waste, which can account for between 35% and 60% of the raw material and are rarely utilized [27,28]. Among these byproducts, the seed accounts for 20% to 25% of the fruit and represents a potential source of biomass, especially due to its high starch content, which can range from 50% to 78% on a dry matter basis [29]; its valorization could facilitate its reintegration into the production chain and contribute to circular economy strategies.

Similarly, taro (*Colocasia esculenta*), known locally as uncucha in Peru, is a perennial tuber crop native to tropical and subtropical regions, with a cultivation history spanning over 9000 years and a wide distribution across diverse agroecological environments [30].

Despite its historical importance as a food crop, taro remains relatively underutilized, partly due to limited research and development aimed at improving its production, processing, and value-added applications; furthermore, its high perishability constrains postharvest handling and contributes to significant losses during storage [30,31]. In Peru, white uncucha (*Colocasia esculenta* var. *antiquorum*) is cultivated primarily in the Andean–Amazonian region, mainly within small-scale farming systems and for household consumption; however, its functional properties remain largely unexplored. Nevertheless, its corms and cormels are rich in starch, with reported contents ranging from 70% to 80% on a dry-weight basis, making it a valuable resource for various formulation systems [32].

Starches extracted from these raw materials exhibit characteristics comparable to those from commercial sources, capable of imparting firmness, elasticity, strength, and stability to the systems into which they are incorporated [33,34,35,36]. Consequently, the objective of this research is to characterize the inherent properties of native starches extracted from Bellaco plantains, Criollo mangoes, and white taro (var. antiquorum) through compositional, colorimetric (CIELab), morphological (SEM and PSA), structural (XRD and FTIR-ATR), hydrothermal (swelling capacity, solubility, and water absorption), rheological (paste-forming and dynamic properties), and thermal (DSC and TG/DTG) analyses; thereby enabling an understanding of the relationships between the structure and function of these starches, as well as promoting the valorization of underutilized plant resources for sustainable applications.

## 2. Materials and Methods

### 2.1. Samples and Materials

Banana, mango, and taro samples were purchased at the Mercado Modelo in Quillabamba and sourced from agricultural farms located in the districts of Quellouno and Echarati, La Convención Province, Cusco Region, Peru. Plantains of the ‘Bellaco’ variety and mangoes of the ‘Criollo’ variety were used, both harvested while still unripe, while the corms of white taro (var. ‘antiquorum’) were obtained at commercial maturity. All plant materials were selected based on quality criteria, transported in plastic baskets, and subjected to a washing and disinfection process by immersion in a 200-ppm sodium hypochlorite solution for 3 min, followed by rinsing with running water until they were ready for further processing. The reagents used in the study were of analytical grade. The materials, equipment, and instruments used for the analyses and experimental procedures were provided by the laboratories of the Department of Food Engineering at the National Intercultural University of Quillabamba, Peru (UNIQ), and by the Center for Agri-Food Technology (CTA) of the Department of Food Engineering, together with the Multi-User Laboratory Complex (C-LABMU) at the State University of Ponta Grossa, Brazil (UEPG).

### 2.2. Starch Isolation

The starch was extracted using the hot extraction method, following the procedure proposed by [37,38,39] with some modifications. The plantains and taro were peeled, cut into small pieces, and immersed in a 0.3% (*w*/*v*) citric acid solution to prevent browning. The mangoes were peeled to expose the endocarp, and the cotyledons were separated by making a cut along the sides and gradually opening them. The resulting cotyledons were washed with drinking water to remove any remaining residue and impurities. The banana pulp, mango cotyledons, and peeled taro corms were homogenized in a Skymsen LAR-25LMB industrial blender (Brusque, Brazil), at 3500 rpm for 5 min with distilled water in ratios of 1:1 (*w*/*v*), 1:3 (*w*/*v*), and 1:5 (*w*/*v*), respectively. The resulting mixture was poured through a No. 60 (250 µm) stainless steel sieve from ELE International (Milton Keynes, UK), and continuously sieved using woven wire sieves from GRAN TEST (Bogotá, Colombia), with decreasing mesh sizes: No. 80 (180 μm), No. 100 (150 μm), and No. 120 (125 μm), with continuous addition of distilled water. The resulting suspension was allowed to settle for 4 h, 8 h, and 24 h at 18 °C to allow for adequate starch sedimentation while maintaining controlled conditions and minimizing potential microbial and enzymatic activity during the prolonged sedimentation. The supernatant was slowly removed, and the sediment was centrifuged at 3500 rpm for 15 min in an Ortoalresa Digtor 22 centrifuge (Daganzo de Arriba, Spain), in equal quantities of 350 g per tube. The resulting sample was placed on stainless steel trays, separating the pigmented fraction from the starch; it was then dried at 40 °C for 24 h in a Universal Memmert model UF160plus (Schwabach, Germany). After drying, the starch was pulverized using a RETSCH model Ultra ZM200 centrifugal pulverizer (Eibar, Spain), at 8000 rpm for 30 s, sieved through a No. 170 mesh (90 μm) (Gran Test, Bogotá, Colombia), and stored in airtight LDPE bags in a dry environment at 4 °C until further analysis.

### 2.3. Starch Characterization

#### 2.3.1. Starch Composition

The chemical determination of native starches was performed according to methods established by the Association of Official Analytical Chemists (AOAC) [40]. Method 942.05 was used for ash content, method 954.01 for protein content, and method 920.39 for lipid content of the samples [41]. Total carbohydrate content was calculated by difference, by subtracting the sum of the percentages of moisture, ash, protein, and lipids from 100% (*w*/*w*). The value obtained included both available carbohydrates and dietary fiber present in the samples [42].

##### Moisture Content

Moisture content of the extracted starches was determined using an Exact Scale PREMIUM DH-EX moisture analyzer (Cabot, AR, USA), based on the methodology described by [39,43]. Approximately 1.5 g of starch was placed in the sample pan, and the sample was dried at 105 °C until a constant mass was reached.

##### Ash Content

The ash content was determined using the gravimetric method involving carbonization and muffle furnace calcination. Five grams of sample were weighed into porcelain crucibles and subjected to initial carbonization on a hot plate. Subsequently, the crucibles were placed in a muffle furnace preheated to 550 °C for 5 h until a white to light gray mineral residue was observed. After calcination, the crucibles were allowed to cool in a glass desiccator and were finally weighed on a SHIMADZU AUY220 analytical balance (Kyoto, Japan).

(1) $$Ash\left(\%\right)=\frac{weight\;of\;ashes}{sample\;weight}\times 100$$

##### Protein Content

The crude protein content was determined using the Kjeldahl method. Then, 0.5 g of starch sample was weighed into Kjeldahl tubes. Then, 1.5 g of a catalytic mixture (CuSO_4_ + K_2_SO_4_) and 6 mL of analytical-grade concentrated sulfuric acid (H_2_SO_4_) were added. The samples were placed in a digestion block and heated from 50 °C to 400 °C until an emerald-blue solution was obtained. After cooling, 10 mL of distilled water was added to the digestate. Distillation was carried out in a MARCONI model MA036 nitrogen distiller (Piracicaba, Brazil), with 50% (*m*/*v*) NaOH added to the tube in situ. The resulting solution was collected in a 2% (*m*/*v*) boric acid (H_3_BO_3_) solution containing 5 drops of mixed indicator in the previously prepared receiving flask. Finally, the distillate was titrated with 0.1 mol L^−1^ hydrochloric acid (HCl), with a correction factor (f) of 1.0638, until the color of the sample turned pinkish-orange.

(2) $$N\;\left(\%\right)=\frac{V\times N\times f\times 14\times 0.1}{sample\;weight}\times 100$$

(3) $$Protein\left(\%\right)=N(\%)\times 6.25$$

where *N* (%): Percentage of nitrogen; *V*: volume of HCl used in the titration; *N*: normality of HCl; and f: HCl correction factor.

##### Lipid Content

Then, 5 g of a dry, sieved starch sample was placed in filter paper extraction cartridges (Unifil, diameter = 15 cm). The cartridge was secured on both sides and placed in the Soxhlet extractor. It was positioned above a 250-mL flask containing n-hexane (analytical grade, PA; NEON, CAS No. 110-54-3) as the solvent and mounted together with the condenser assembly on a universal stand. The extraction was carried out at 150 °C for 4 h with a condenser flow of water at room temperature entering and exiting the system. The solvent and lipid mixture was collected in the flask, while excess solvent remained inside the extraction unit; the excess was removed along with the sample cartridge, and the evaporation process was continued. The flask was transferred to a drying oven (RSA) at 105 °C for 12 h and allowed to cool for 30 min inside a glass desiccator, ensuring that the hot air was allowed to escape.

(4) $$Lipids\;\left(\%\right)=\frac{lipids\;weight}{sample\;weight}\times 100$$

##### Amylose Content

The amylose percentage was quantified using the potentiometric method with a Titrino Plus automatic titrator (Metrohm, Herisau, Switzerland) equipped with a measuring electrode, in accordance with [42]. A 100 mg sample of starch, defatted with n-hexane (analytical grade, PA; NEON, CAS No. 110-54-3) for 4 h, was used. The sample mass was adjusted according to the moisture content present in the starch. One milliliter of distilled water and 5 mL of 1 N KOH were mixed for 30 min at room temperature using a VELP Scientific HSC magnetic stirrer (Monza, Italy). Three drops of methyl orange were then added as a pH indicator, followed by 0.5 N HCl as the neutralizing solution and 10 mL of 0.5 N KI to allow the formation of amylose–iodine complexes. The total mass of the sample was adjusted to 100.9 g with deionized water. The electrode was calibrated using prepared EMF solutions, and titration was performed using an iodine titrant obtained by a 1:10 dilution of the stock solution. To calculate the amylose content, the iodine-associated starch value (IAs) was normalized using the amylose affinity index (IA = 0.2). The determination of amylose content was performed in duplicate for each sample.

#### 2.3.2. Starch Color Parameters

The colorimetric parameters of the starches were obtained using a 3nh Model NR110 portable handheld colorimeter (Guangzhou, China). The measurements were based on the International Commission on Illumination (CIE) color system: L* (black-white), a* (red-green), and b* (yellow-blue). Calibration was performed using the white standard provided by the manufacturer (Technology Co., Ltd., Guangzhou, China). The samples were compacted to a flat surface. Based on the results, the WI (whiteness) and YI (yellowness) indices were calculated, as well as the chroma (C*) and hue angle (h°) of the samples [44].

(5) $$WI=100-\sqrt{{\left(100-L^{*}\right)}^{2}+a^{*2}+b^{*2}}$$

(6) $$YI=\frac{142.86{\;b}^{*}\;}{L^{*}}$$

(7) $$C^{*}=\sqrt{a^{*2}+b^{*2}}$$

(8) $$h^{{}^{\circ}}=arctan\left(\frac{b\;*}{a\;*}\right)$$

#### 2.3.3. Starch Particle Morphology

The surface morphology of the starch granules was evaluated using a JEOL Model JCM-7000 scanning electron microscope (Akishima, Japan), following the methodology described in [45,46]. The powdered samples were mounted on aluminum support capsules using double-sided carbon adhesive tape. To ensure electrical conductivity and avoid charging effects, the samples were gold-sputtered using a JEOL Smart Coater cathode sputter coater for 0.5 min. Observations were performed at an acceleration voltage of 10 kV using the Secondary Electron Detector (SED) under vacuum conditions. Different magnifications, ranging from 350× to 3000×, were used according to the morphological characteristics of each sample to obtain representative micrographs and ensure adequate visualization of the starch granules. The morphological images were acquired using the NeoScope software (version 1.620; JEOL Ltd.) integrated into the instrument and analyzed using SMILE VIEW Lab software (version 3.13.0; JEOL Ltd.).

#### 2.3.4. Starch Granule Size

The particle size distribution of the starch samples was determined using an Anton Paar Model 1090 Laser Diffraction Particle Size Analyzer (Graz, Austria), operated in measurement mode for dry samples. Approximately 20–30 g of each starch sample was placed directly into the instrument’s feed system and progressively introduced into the measuring unit during the analysis. The dry dispersion conditions were adjusted individually for each sample. A vibration cycle of 70%, a vibration frequency of 30 Hz, and an air pressure of 1200 mbar were used for the banana starch sample; values of 60%, 45 to 50 Hz, and 2200 mbar for the mango starch sample; and conditions of 50%, 40 Hz, and 2000 mbar for the taro starch sample. The results were expressed as D50 and D90 percentiles, the D [4.3] and D [3.2] particle size parameters, and the Span index. The data were processed using the instrument’s software (version 3.8.2) and the Anton Paar ISO calibration protocol.

#### 2.3.5. Crystalline Profile of Starch

The crystalline structures of the native starches were evaluated by X-ray diffraction (XRD) using a Rigaku Ultima IV X-ray diffractometer (Akishima, Japan) [47]. The diffractograms were obtained using Cu Kα radiation (λ = 1.541 Å) generated at a voltage of 30 kV and a current of 10 mA in continuous scan mode. Measurements were performed over an angular range of 5° to 50° (2θ), with a step size of 0.02° and a scan rate of 10° min^−1^. The system was operated with a divergence slit of 1/2°, a height-limited slit of 10 mm, a dispersion slit of 8.0 mm, and an open receiving slit.

The relative crystallinity (RC) of the starches was determined from the diffractograms obtained using OriginPro 2026 software (OriginLab Corporation, Northampton, MA, USA) by separating the crystalline and amorphous regions [48]. The diffractograms underwent baseline correction, and a smoothed curve was fitted to the diffraction profile to separate the crystalline and amorphous contributions. The area above the smoothed curve was considered the crystalline region, while the area between that curve and the linear baseline was considered the amorphous region. The crystalline peak area and the total diffraction area were integrated over the diffraction angle range of 5° to 50° (2θ). Relative crystallinity was calculated as the ratio of the integrated crystalline area to the total integrated diffraction area (crystalline area + amorphous area).

(9) $$RC\;\left(\%\right)=\left(\frac{crystalline\;area}{total\;area}\right)\times 100$$

#### 2.3.6. Structural Properties Revealed by FT-IR of Starches

The FTIR spectra of the starches were obtained using a Bruker Vertex 70 Fourier-transform infrared (FTIR) spectrophotometer (Ettlingen, Germany), equipped with an attenuated total reflectance (ATR) accessory, as described by [49]. The powdered starch samples were placed directly onto the ATR crystal and analyzed in the mid-infrared (MIR) region over the range of 400–4000 cm^−1^. Each spectrum was acquired from 16 accumulated scans at a spectral resolution of 4 cm^−1^. The spectra were processed in terms of absorbance.

#### 2.3.7. Hydrothermal Properties of Starch

The behavior of starch in excess water was determined using a hydrothermal method, in accordance with the method reported by [44,50,51]. Then, 0.2 g of starch was placed in 10 mL of distilled water in 50-mL Falcom tubes. The samples were heated under constant agitation for 30 min at 60, 70, 80, and 90 °C in a Dubnoff-type metabolic bath with reciprocating agitation (horizontal back-and-forth shaking) while held on a metal rack; subsequently, the samples were cooled in an ice bath and centrifuged at 3400 rpm for 15 min. To calculate solubility, the supernatant was collected and dried at 105 °C in an oven for 24 h, while the sedimented fraction containing swollen starch granules was weighed. The swelling power (SP), solubility index (WSI), and water absorption index (WAI) of the samples were calculated using the following equations.

(10) $$SP\;(g/g)=\frac{swollen\;granules}{sample\;weight-dry\;supernatant}\;$$

(11) $$WSI\;(\%)=\left(\frac{dry\;supernatant}{sample\;weight}\right)\times 100\;$$

(12) $$WAI\;(g/g)=\frac{swollen\;granules}{sample\;weight}$$

#### 2.3.8. Starch Pasting Behavior

The evaluation of the starches’ binding profile was carried out using an Anton Paar Compact Modular Rheometer, model MCR-702e (Graz, Austria), equipped with a starch cell geometry (ST24-2D/2V/2V-30) and an aluminum measuring cup. The procedure was based on the methodology described in [44]. Starch suspensions at 10% (*w*/*w*) were prepared with distilled water, the proportion adjusted according to the moisture content of the starch, using a total sample mass of 20 g. The mixture was homogenized and allowed to stand for 1 h to allow for complete hydration and stabilization of the system. The tests were conducted at a constant shear rate of 160 rpm. The thermal profile consisted of an initial holding stage at 50 °C for 2 min, followed by heating from 50 to 95 °C, an isothermal phase at 95 °C for 5 min, a cooling ramp from 95 to 50 °C, and a final isothermal stage of 2 min at 50 °C, at a rate of 6 °C min^−1^ for a total duration of 23 min. The parameters obtained were processed using Reocompass© software, version 1.30.999.

#### 2.3.9. Dynamic Properties of Starch

The dynamic properties of the starches were evaluated by means of a frequency sweep using an Anton Paar Model MCR-702e Compact Modular Rheometer (Austria), in accordance with the method reported by [52]. The starch gels were prepared from 10% (*w*/*w*) starch suspensions. Measurements were performed using parallel plates (PP50) with a diameter of 50 mm and a gap of 1 mm, at a constant temperature of 30 °C. The frequency sweep was conducted over an angular range (ω) of 0.1 to 100 rad s^−1^ with a constant strain (γ) of 1%. The storage modulus (G′) and loss modulus (G″) were determined, as well as the loss factor (tan δ). The results were expressed at an angular frequency of 1 rad s^−1^.

#### 2.3.10. Calorimetric Analysis of Starch

The thermal properties of gelatinization were analyzed using a TA Instruments DSC 2500 differential scanning calorimeter (DSC), New Castle, DE, USA. Parameters were set according to [41]. A starch suspension was prepared in a 1:3.5 (*w*/*v*) ratio. Ten milligrams of the suspension were weighed into Tzero Pan aluminum capsules, which were hermetically sealed using a Tzero press from TA Instruments and allowed to stand for 1 h prior to analysis. A temperature ramp from 30 to 120 °C was used at a constant rate of 5 °C min^−1^ with a high-purity nitrogen flow (Peak Scientific model NG3000A-220-EU, Inchinnan, UK) of 25 mL min^−1^, using an empty reference crucible. TRIOS™ software version 5.4.0.300 was used to determine the onset temperature (*T*_o_), peak temperature (*T*_p_), end temperature (*T*_c_), and gelatinization enthalpy (△*H*).

#### 2.3.11. Thermogravimetric Analysis of Starch

The thermal stability and degradation of the starches were analyzed based on [53], using a TA Instruments TGA 5500 thermogravimetric analyzer (USA). Ten milligrams of powdered sample was weighed onto aluminum pans. The analysis was performed under a high-purity nitrogen atmosphere (Peak Scientific model NG3000A-220-EU, United Kingdom) at a flow rate of 25 mL min^−1^, using a controlled heating program from 25 to 700 °C at a constant rate of 10 °C min^−1^. The thermogravimetric (TG) and derivative thermogravimetric (DTG) curves were obtained using TRIOS™ analysis software (version 5.4, TA Instruments), with DTG representing the first derivative of the TG curve with respect to temperature.

#### 2.3.12. Statistical Analysis

Statistical analysis was performed using the data obtained from the experimental replicates described in each methodology. A one-way ANOVA was applied to compare samples across the different characterization techniques, while the data from the hydrothermal analysis were evaluated using a two-way ANOVA with the OriginPro 2026 software (OriginLab Corporation, USA). Multiple comparison tests (Tukey) were performed to specifically identify different groups among the data means, using a significance level of *p* < 0.05 and a 95% confidence level.

## 3. Results

### 3.1. Starch Composition

The proximate analysis and amylose content determination of the samples, summarized in Table 1, revealed statistically significant variability in the intrinsic chemical composition of the extracted native starches. Quantifying and understanding these parameters is a critical indicator of purity and interaction between the polysaccharide chains that condition the supramolecular architecture of starch, which influences its applicability, allowing the interpretation of its rheological response and the thermal transitions associated with gelatinization. The observed variations can be attributed to the botanical nature of the starches studied.

The water content of the starches studied ranged from 7.30% to 7.90%, values below the reference limit (<12%) established for native or modified starches to prevent caking and structural deterioration [54]. Associated with greater stability during storage and lower susceptibility to microbiological degradation [55], allowing the granule to maintain its semicrystalline structure, degree of gelatinization, and retrogradation [56].

The ash content ranged from 0.1% to 0.28%, indicating adequate removal of residual mineral components during extraction [57]. The significantly higher variation observed in TCS can be attributed to the intrinsic composition of the source plant tissue, which is influenced by growing conditions [58,59]. This falls within the range reported for *Colocasia esculenta* starches (0.22–0.31%) in similar varieties [60,61]. According to [62], the availability of minerals from biomass ash can affect the internal structure of the pellet, altering its properties during heating and cooling and influencing its behavior in complex incorporation systems [63].

The protein and lipid content varied among the starches evaluated. BPS had protein (1.13%) and lipid (0.11%) values similar to those previously reported in [64], where ranges of 1.28 to 1.6% for protein and 0.12 to 0.7% for lipids, On the other hand, lower protein values (0.16–0.30%) and lipid values (0.15–0.42%) were recorded for starches isolated via alkaline extraction [65], indicating variability associated with the plant species and the extraction conditions used. As reported [66,67], in, saline, alkaline, and acidic treatments have shown greater removal efficiency by promoting the solubilization of proteins from the granular surface and the dissociation of lipid complexes. In MCS, protein and lipid contents of 1.93% and 1.10%, respectively, were observed, which are higher than those reported in [36], where values ranging from 0.77% to 0.84% for protein and from 0.09% to 0.54% for lipids; this difference is related to the maturity stage of the plant material studied. The most significant difference observed in MCS can be explained by its structural complexity, the biological function of the tissue, and cultivation conditions, with high values of up to 5.7% protein and 9.3% lipids reported, exceeding those recorded for conventional starches such as corn and potato [68]. For its part, TCS exhibited respective amounts of 1.69% and 0.10% for proteins and lipids, close to those reported in [69], where a protein content of 1.46% and a lipid fraction of 0.43% were described. The relatively higher protein content may be attributed to the presence of the mucilaginous matrix characteristic of taro. Due to its viscous and polysaccharide-rich nature, this matrix may hinder the separation and removal of non-starch components during starch isolation, favoring the retention of proteinaceous material and, consequently, its residual presence in the isolated starch. According to [70,71], the presence of these components influences starch gelatinization, since proteins can act as a physical matrix around the granule, limiting its interaction with water, the mobility of the polysaccharide chains, and the release of amylose, whereas lipids promote the formation of inclusion complexes with amylose (amylose-lipid) that affect the viscosity and retrogradation properties of starch, reducing hydration and the fraction of amylose available for leaching.

Amylose is the linear fraction that plays a decisive role in the functional properties of starch [72]. In this study, the amylose content recorded for BPS, MCS, and TCS was 25.92%, 21.97%, and 16.83%, respectively. Similar values (21.97–55.46%) [65] and (19.32–26.35%) [73] have been reported for green plantain starches from different species and cultivars of the genus *Musa*; values in the same range (25.82–27.98%) [74] for starches from green mango seeds of the ‘Keitt’, ‘Palmer’, ‘Parwin’, and ‘Tommy Atkins’ varieties; and similar values (18.33%) [75] and (13.2–16.2%) [76] for starches from the esculenta and antiquorum varieties of taro. The significantly higher amylose content observed in PBS and MCS is associated with greater firmness and gel strength, a greater tendency toward retrogradation, and lower granule swelling capacity [77], contributing to the formation of dense, compact structures with greater mechanical strength [78]. These values are comparable to those reported for starches from conventional sources such as corn starch (14.85–25.16%) [79], yellow sweet potato (16.97–26.83%) [42], and cassava (13.2–29.5%) [80] depending on the variety, growing conditions, and type of isolate [75].

### 3.2. Starch Color Parameters

The color parameters listed in Table 2 represent an important criterion for the evaluation and specific applicability of starch [81], allowing for the identification of differences in its purity and in the presence of compounds associated with the source matrix. In this context, the L*, a*, and b* parameters, together with the derived indices WI, YI, C*, and h°, provide a description of the lightness, hue, and saturation of the samples studied; comparing these values allows for the interpretation of the significant differences found among the starches studied.

PBS had a lower L* value (84.53), indicating a higher presence of non-starch compounds associated with the matrix, such as polyphenols and residual pigments. This result is similar to that reported in [39] for plantain starches from the *Musa* AAA and ABB genotypes, which had L* values (82.67 and 89.57) and a* values (2.83 and 1.61) and b* values (10.92 and 9.03), and is lower than those described in [82], where L* values (ranging from 92.55 to 95.71) were reported, along with negative a* values (ranging from −11.89 to −10.75) and b* values (5.42 and 6.26). Likewise, it has been documented that modification treatments such as oxidation can increase the brightness of plantain starch within a range of 82.4 to 96.74 [83]. This reveals variability in the optical properties depending on the genotype and the conditions under which the starch was obtained. The positive values of a* (4.10) and b* (12.40) indicate a tendency toward reddish hues with a predominance of yellow tones; and together with the values of C* (13.06) and h° (71.69), they confirm greater color intensity and a greater contribution of the red component within the spectrum, supported by the lower WI index (79.75) and higher YI index (20.96).

Compared to plantain starch, MCS had a lighter appearance with a slightly creamy color (L* = 90.04), consistent with seed starches from different cultivars (L* = 90.54–93.56) [36]. The a* (1.47) and b* (11.72) values indicate a dominant hue on the yellow axis with moderate saturation, consistent with the values of h° = 82.85, C* = 11.82, and YI = 18.60. However, Refs. [84,85] reported lower values of L* = 82.68 and 86.23, attributed to the higher carotenoid content in the seed associated with this lipid fraction. The WI value of 84.54 recorded for this starch indicates adequate light reflectance and visual purity; according to [85], values close to WI = 83 have been associated with products having favorable optical characteristics.

In this study, TCS exhibited the highest lightness (L* = 98.30) and whiteness index (WI = 96.56), indicating high visual purity and minimal interference from chromophore compounds, comparable to commercial cornstarch (L* = 98.02) [82], although higher than those reported in [76] for esculenta and antiquorum taro varieties (L* = 93.3 and 94.4) and in [86] for native taro starch (L* = 94.2) and starch modified by acid and ultrasound treatment (L* = 94.8–95.9), suggesting higher optical quality of the starch studied. The a* values close to zero (−0.07) and the low b* value (2.99) reflect a neutral hue close to white with a low tendency toward yellow regions, confirmed by the lower values recorded for the YI indices: 4.35 and C* = 2.99. The optical values of TCS are important for the formulation of food products without visual alterations, thereby enhancing their final organoleptic acceptability [87].

### 3.3. Starch Particle Morphology

**Figure 1:**
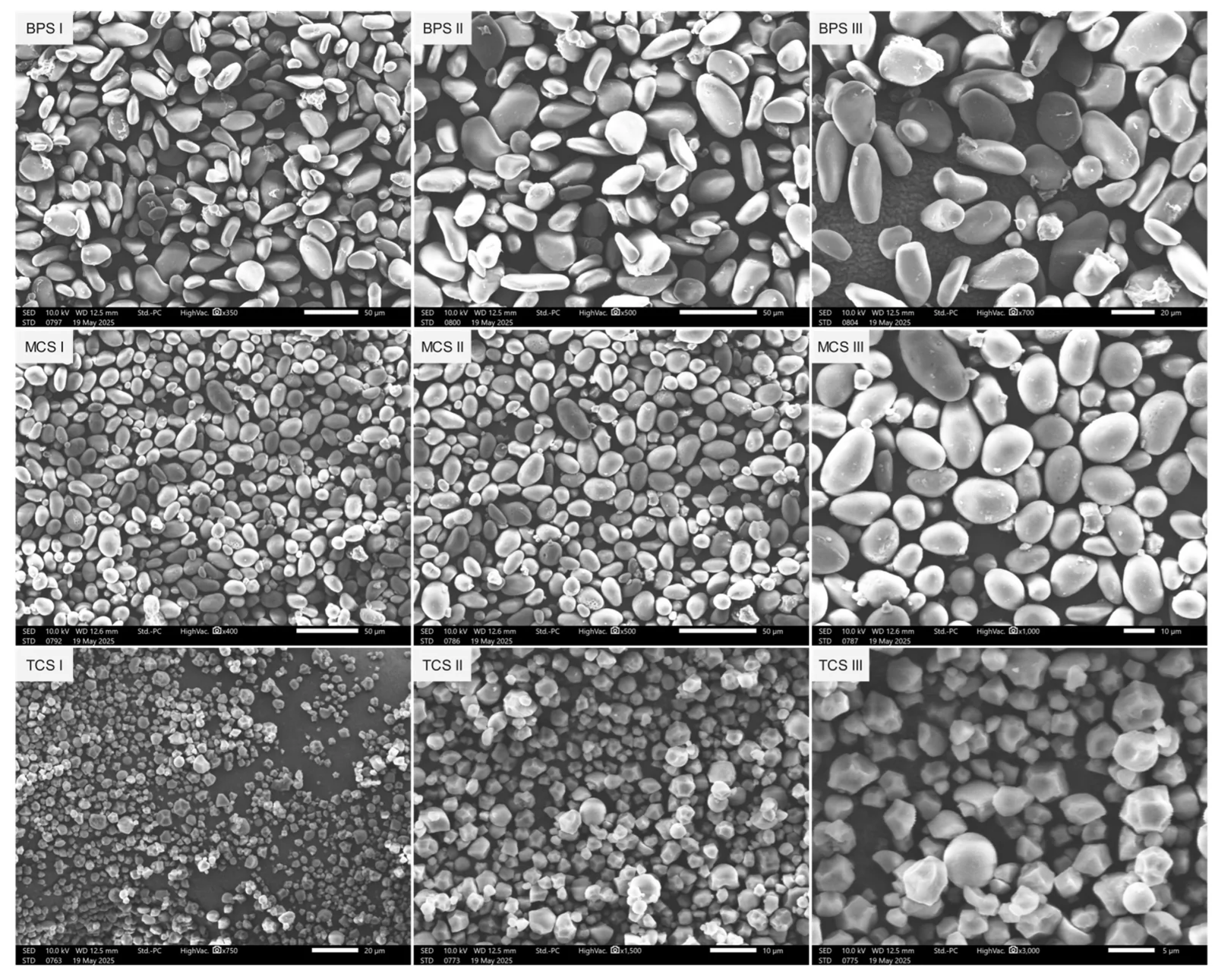
SEM micrograph of native starch of BPS: banana pulp starch; MCS: mango cotyledon starch; TCS: taro corm starch. Photographs with magnifications of 350×, 500× and 700× (BPS I, II, III); 400×, 500× and 1000× (MCS I, II, III); 750×, 1500× and 3000× (TCS I, II, III).

The surface microstructure of the native starch particles evaluated revealed distinctive morphological patterns (Figure 1) related to the botanical origin of the samples. The PBS granules were oval to elongated ellipsoidal in shape and larger in size, with rounded ends and a flattened/lenticular appearance; whereas the granules corresponding to MCS exhibited a more diverse morphology dominated by ovoid, elliptical, and rounded shapes with a more compact distribution. In contrast, TCS samples contained polygonal, subspherical, and irregular granules that were considerably smaller in size.

The morphological characteristics observed in the starches were consistent with those reported in the literature, with variations attributable to the genotype analyzed. For starches from the AA banana cultivar, irregular shapes with a flattened tendency were described; in AAA cultivars, elongated and spherical granules were observed, while in AAB and AAAB cultivars, rod-like, elongated, and oval structures were found [65,88]. Similarly, in seed starches from different cultivars of *Mangifera indica*, populations consisting of oval and spherical granules are commonly found [36]. In taro starches, on the other hand, more irregular morphologies were reported, with a wide range of shapes ranging from polyhedral, polygonal, and ellipsoidal to rounded and spherical [31].

The surface of the evaluated granules showed a continuous structure, with no evidence of significant damage associated with the extraction process. However, small surface pores with sparse distribution were observed only in MCS on some granules. Previous studies on mango seed starch have reported the presence of pores, grooves, and rough folds [36,74,89], which may be attributed both to intrinsic characteristics of the granular structure and to modifications that occurred during starch isolation. According to [65], uniformity and the absence of surface roughness may be related to a more compact and ordered arrangement of the granule’s molecular chains, conferring lower external susceptibility and limiting structural defects. In contrast, starches with highly porous surfaces and physical damage tend to exhibit a greater affinity for water, leading to changes in granular hydration during gelatinization [90]. Additionally, Ref. [91] reports that aggressive and prolonged mechanical treatments can induce cracks, fractures, the formation of grooves, roughness, re-agglomeration of fragments, and loss of the granule’s internal organization.

### 3.4. Starch Granule Size

The particle size parameters of the starches summarized in Table 3 show significant differences among the samples, indicating variations in particle size.

The significantly elevated values for the D50 and D90 percentiles in BPS (22.40 μm and 41.50 μm) indicate a distribution shifted toward larger sizes, with a greater contribution from higher-volume particles in the coarse fraction of the distribution, consistent with the reported higher mean diameter (D[4,3] = 24.37 μm). This is consistent with the range described for starches from different genomic groups of the genus *Musa* (AAA, AA, and AAB) in [92], with average particle sizes between 21.73 μm and 24.67 μm and size distributions ranging from 5.61 to 69.61 μm, and with the parameters reported in [93], including D50 = 16.05 to 25.52 μm; D90 = 30.47 to 44.87 μm; and D[4,3] = 18.98 to 32.22 μm.

Meanwhile, MCS showed intermediate values (D50 = 13.48 μm, D90 = 28.11 μm, and D[4,3] = 15.21 μm), suggesting a balanced particle size distribution, comparable to that reported for green mango seed starches, with D[4,3] values ranging from 19.75 to 25.33 μm [74], and size distributions ranging from 10.9 to 27.2 μm [68]. In addition to variety and botanical origin, it has been documented that the stage of maturity of the raw material exerts a significant influence on starch granule size [94]. Likewise, the method used for starch extraction is a determining factor in the size distribution of the resulting particles [95].

In contrast, TCS exhibited the lowest values (D50 = 4.43 μm, D90 = 8.01 μm, and D[4,3] = 6.64 μm), revealing a distribution skewed toward smaller, finer particles. According to [31], the size distribution of taro starch granules (*C. esculenta* and *C. antiquorum*) is generally smaller compared to granules from tuberous roots such as cassava (5 to 40 μm) [80] and tubers such as the potato (15 to 110 μm) [96], with reported sizes ranging from 0.05 μm to 20 μm and a predominance of values below 10 μm in most of the cultivars analyzed, depending on the botanical origin and the measurement method used.

The particle size distribution exhibited a unimodal profile, characterized by a well-defined main peak in each starch evaluated and the presence of slight shoulders or secondary size subpopulations. However, statistical analysis revealed no significant differences (*p* > 0.05) in the surface-area-weighted mean diameter (D[3,2] = 1.34–4.50 μm) or distribution width (Span = 1.63–2.06), suggesting an overall relatively uniform and comparable particle size distribution among the starch samples [97]. According to [98], intensive extraction processes may compromise the structural integrity of starch granules, potentially resulting in modifications in both granule size and particle size distribution.

**Figure 2:**
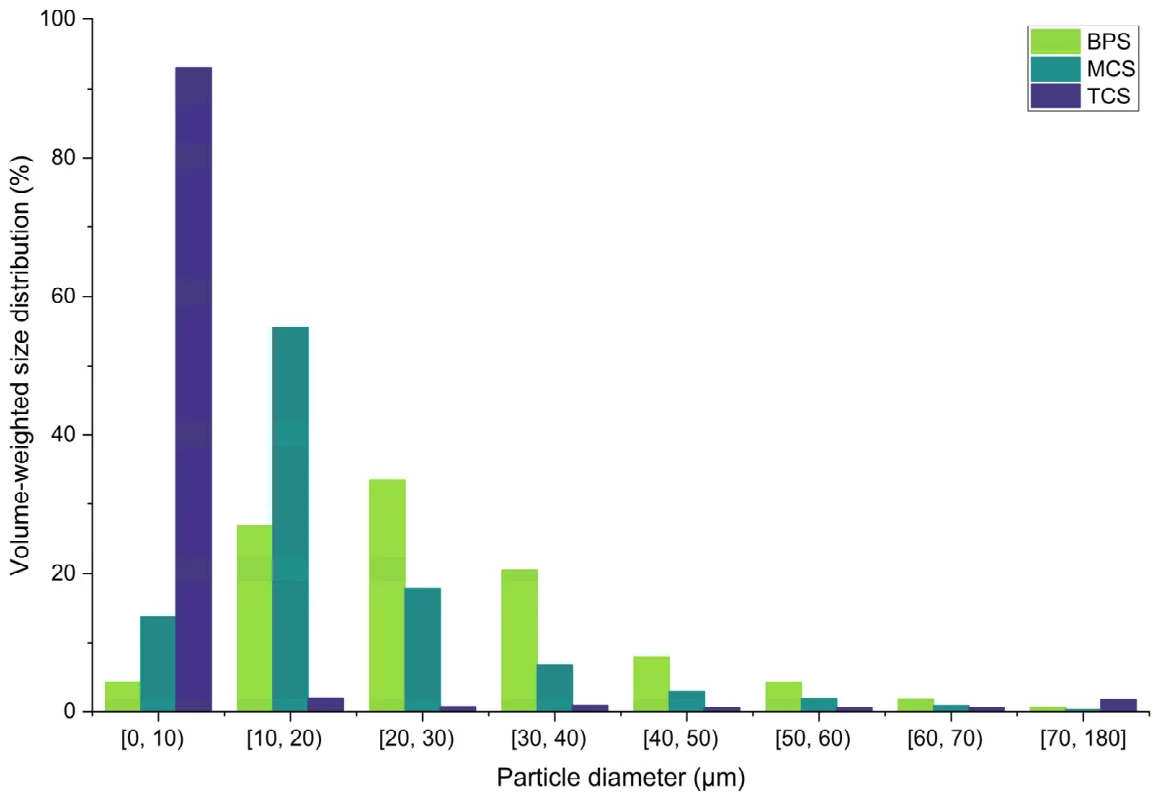
Volumetric particle size distribution by diameter intervals of BPS: banana pulp starch; MCS: mango cotyledon starch; TCS: taro corm starch.

**Figure 3:**
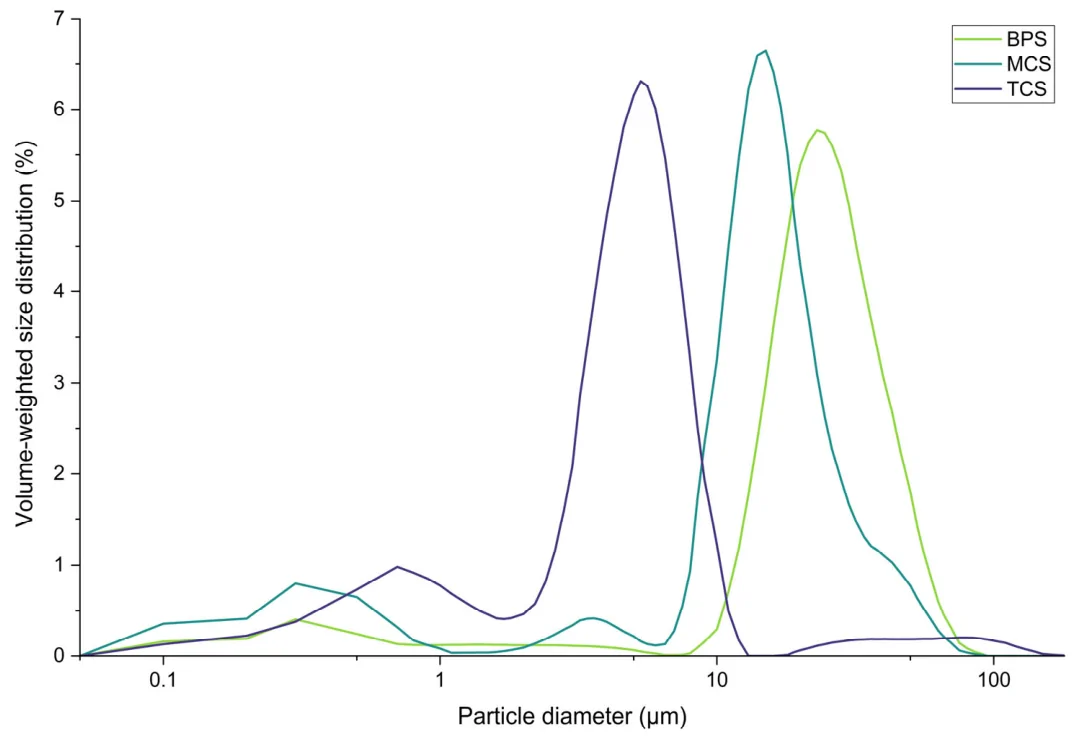
Granule size distribution of BPS: banana pulp starch; MCS: mango cotyledon starch; TCS: taro corm starch.

The analysis by particle size range presented in Figure 2 confirmed the trends observed in Figure 3 and in the particle size parameters (Table 3). TCS showed a predominance of more than 90% of the total particle volume in the 0–10 µm range, whereas MCS was concentrated in the 10–20 µm range (>50%), and BPS showed a predominance in the 20–30 µm range (>30%), confirming a gradient in size distribution among the starches studied. These differences in particle size can be attributed to genetic variations and developmental conditions, which affect starch biosynthesis, amylose content, and granular morphology [93]. Furthermore, the variation observed in starch particle size plays an important role in starch properties and functionality, broadening its utility in various systems. As indicated in [65,84,99], particles with larger diameters are suitable for use in systems as thickeners and gelling agents, as well as in baked goods, promoting controlled hydration and the formation of stronger, more stable networks with greater expansion capacity. Conversely, smaller-sized fractions are generally preferred in applications where rapid gelatinization, reduced retrogradation, and greater homogeneity are desired, due to their higher surface area per volume and better dispersion.

### 3.5. Crystalline Profile of Starch

The crystalline arrangements commonly identified in native starches are of the pure polymorphic type (A and B) and mixed polymorphic type (C), with simultaneous A/B crystals, as determined by X-ray diffraction (XRD) through the scattering of X-rays by the ordered molecular fractions of the starch into specific angular patterns (2θ) [31]. Their intensity and position are closely correlated with intrinsic structural factors, applied treatments [100] or the availability of amylose with accessible lipid fractions [101].

**Figure 4:**
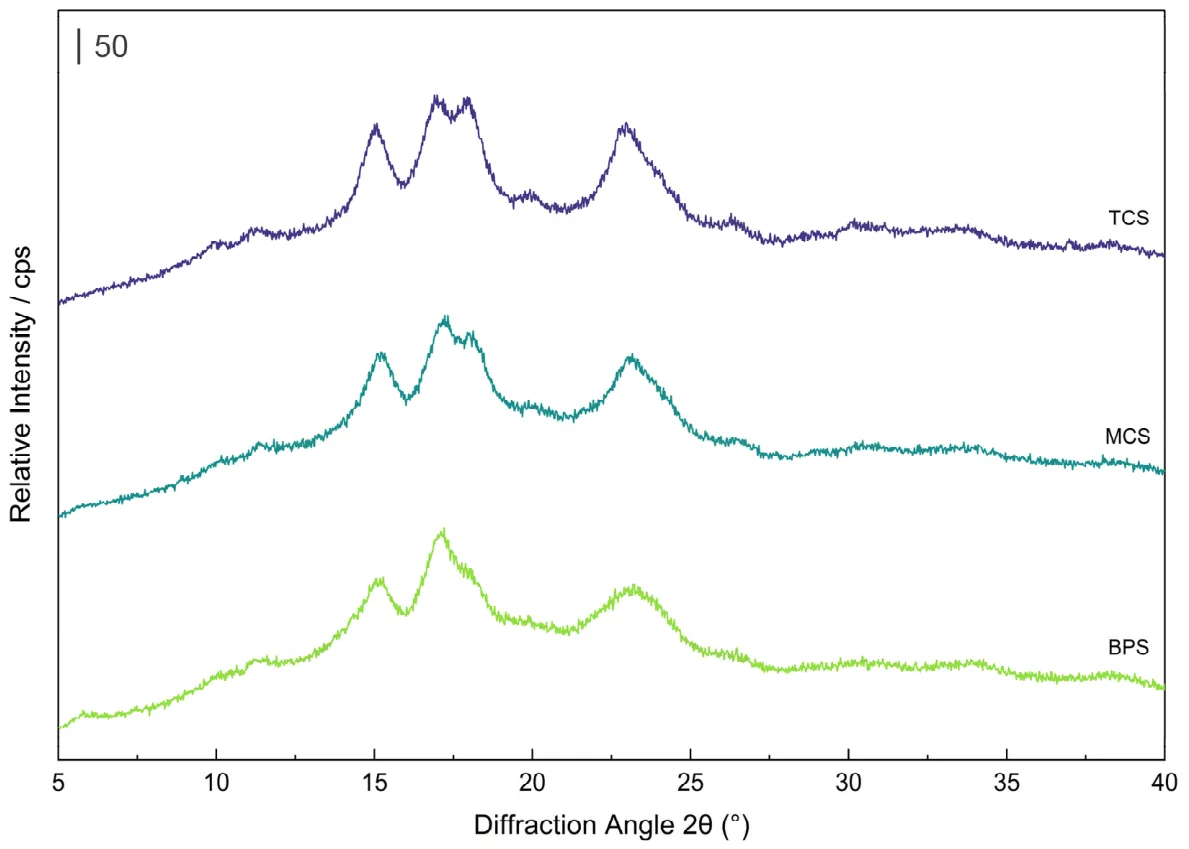
Crystallinity patterns of banana pulp starch (BPS), mango cotyledon starch (MCS), and taro corm starch (TCS).

The X-ray diffraction patterns of the starches (Figure 4 and Table 4) showed typical semicrystalline structures. MCS and TCS exhibited characteristic Type A diffraction reflections with isolated signals (15.2° and 23.16°; 15.08° and 22.96°) and overlapping peaks (17.22° and 18.12°; 16.98° and 17.94°), respectively. This polymorphism is associated with a higher molecular packing density and low intralaminar hydration [36] consisting of short side chains and closed double-helix branches, in contrast to the type B hexagonal pattern, which has a more widely spaced structure and longer chains [102,103]. This is commonly reported in cereal starches [100,104], although also described in certain cassava starches [104]. In contrast, BPS exhibited a weak reflection at 2θ = 5.78°, characteristic of type B, whose coexistence with type A peaks suggests a type C crystalline structure.

These differences in crystalline polymorphism suggest that the supramolecular organization of the granules could help modulate their interaction with water and their response during processing, with implications for functional properties relevant to their technological applications [105]. According to the studies compiled in [106], type A starches may exhibit greater gelatinization capacity and viscosity development—characteristics of interest in sauces, baked goods, firm-textured foods, and film-forming materials; however, they tend to be more susceptible to digestive enzymes. On the other hand, the characteristics associated with polymorph B may favor a structure that is less accessible to enzymes and slower digestion, related to the formation of resistant starch, which could be of interest for nutritional applications. Therefore, pattern C could exhibit intermediate or variable behavior depending on the relative proportion of polymorphs A and B that comprise it, with potential for applications in functional foods, thickening systems, or products where the goal is to combine technological and nutritional functionality. However, previous studies have suggested that crystalline polymorphism, by itself, does not determine starch functionality; rather, this results from its interaction with other physicochemical and functional properties of the granule [107].

The diffraction intensity and the degree of crystallinity reflect differences in the structural ordering and molecular organization of the granules, which are influenced by the content, size, and interaction of the amylopectin chains, as well as by the number and dimensions of the starch’s crystalline domains [108]. As shown in Figure 4, TCS exhibited higher relative intensity of the diffraction peaks, indicating greater crystalline order [109], compared to MCS and BPS, which is consistent with its higher relative crystallinity (RC; 36.68%) and lower amylose content [110]. This higher degree of order could limit the diffusion of hydrolytic agents into the interior of the granule, contributing to greater structural strength [104,111]. In contrast, the lower peak resolution of BPS and its lower relative crystallinity (29.67%) could be associated with its higher amylose content, which would favor a higher proportion of amorphous regions, lower molecular packing, and greater mobility of the polymer chains, facilitating water penetration [112].

The relative crystallinity reported for taro starch varies widely, ranging from 27% to 45%, depending on the botanical source studied. Furthermore, the loss of intensity in the diffraction peaks has been associated with alterations in the crystalline order of the starch, resulting from the breaking of hydrogen bonds and the displacement of the amylopectin double helices [31]. Similarly, banana starch exhibits crystallinities ranging from 28% (21.3% amylose) [113] to 57.3% (16.36% amylose) [114]. Likewise, mango seed starch exhibits RC values between 35.4% and 38.3% in the ‘Kuppi’ and ‘Chausa’ varieties, with amylose contents of 33.6% and 28.8%, respectively [115], confirming the inverse relationship between the degree of crystallinity and the amount of amylose present in the granule.

### 3.6. Structural Properties Revealed by FT-IR of Starches

**Figure 5:**
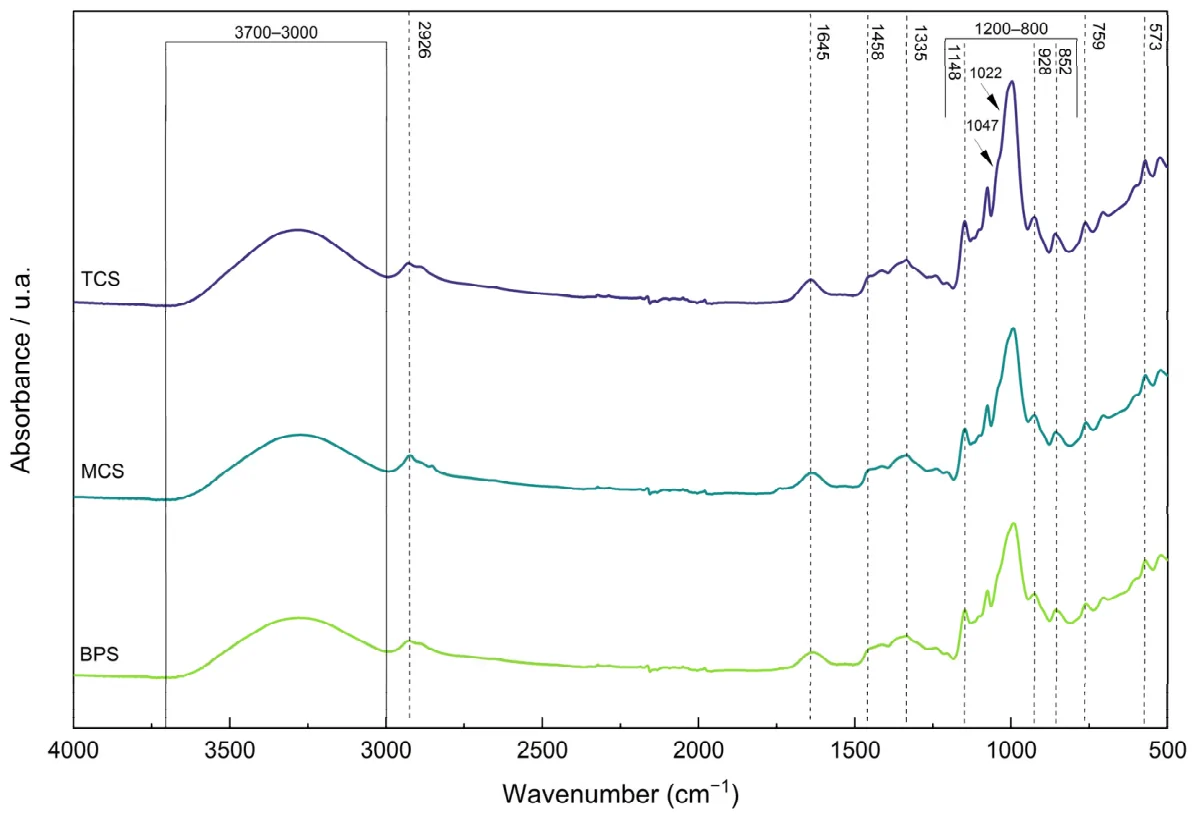
FTIR-ATR spectral profiles of banana pulp starch (BPS), mango cotyledon starch (MCS), and taro corm starch (TCS).

The attenuated total reflectance Fourier transform infrared (FTIR-ATR) spectra of the three starches are shown in Figure 5. The samples revealed functional groups characteristic of polysaccharide structures typical of native starchy systems. Differences in band intensity are observed among the samples, indicating variations in the degree of structural order and in the intermolecular interactions present.

A broad band was observed between 3700 and 3000 cm^−1^, corresponding to the stretching of the hydroxyl (O-H) groups in the glucose units [116]. The higher intensity observed in TCS could be related to a greater presence of intra- and intermolecular hydrogen bonds within the granular structure of the starch. Likewise, the band observed around 2900 and 2960 cm^−1^ is attributed to stretching vibrations of the C-H_2_ bonds in the glucosidic units [116,117], while the signal located at approximately 1645 cm^−1^ is associated with the bending of the H-O -H bond of water adsorbed onto the starch, and the peak located around 1458 cm^−1^ reflects the interactions between water molecules and the amorphous starch matrix via hydrogen bonds [116,118]. Furthermore, the band at 1335 cm^−1^ is related to the vibration of C-OH and CH_2_ groups present in the starch structure [109].

TCS and MCS showed greater intensity in the bands in the region between 1200 and 800 cm^−1^ (fingerprint region), associated with the skeletal vibrations of the glucopyranose ring present in the structural units of amylose and amylopectin, C-O-H, C-O, and the glycosidic bond C–O–C [117]. The signal located around 928 cm^−1^ is related to vibrations of the α-(1 → 4) bonds of the D-glucopyranosyl ring that constitute the main chains of the polysaccharide [101], while bands near 852 cm^−1^ and 759 cm^−1^ are attributed to deformation vibrations of C–H and CH_2_ groups, as well as to vibrations of the carbon backbone (C–C) [109,119]. Furthermore, the band at 1148 cm^−1^ can be attributed to the stretching of ether groups present in the glycosidic bridges of starch, while the peak at 573 cm^−1^ is associated with the movement of the C–C–C bond, allowing the identification of the starch component of the sample; these peaks were reported in Ref. [120] in the regions of 1155 and 580 cm^−1^ for Peruvian sweet potato starches.

The higher intensity in TCS may be associated with greater local organization of the amylopectin double helices and a more developed short-range structural order, consistent with the nature of starches with type A diffraction patterns [121], which have more compact and denser structures (Figure 4). In contrast, BPS exhibited a lower relative intensity in this spectral region, which could be associated with a lower molecular packing density and a more heterogeneous distribution of crystalline and amorphous domains, consistent with the sample’s crystalline type (C).

### 3.7. Hydrothermal Properties of Starch

The values presented in Table 5 reveal a statistically significant interaction between temperature and the type of starch analyzed, indicating a marked dependence between the two factors. This allows us to interpret how the functional characteristics of starch respond to different temperature conditions in systems with excess water.

**Figure 6:**
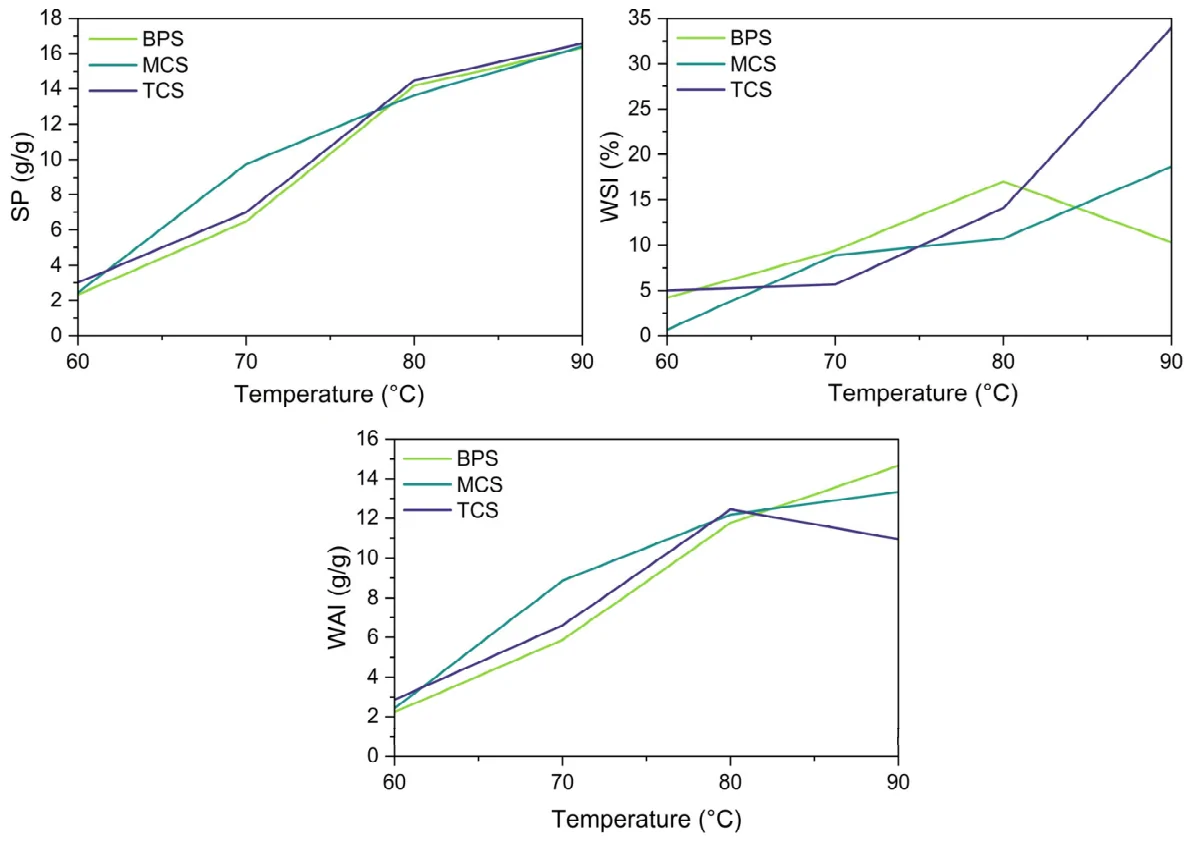
Profile of SP: Swelling Power; WSI: Solubility; WAI: Water Absorption of BPS: banana pulp starch; MCS: mango cotyledon starch; TCS: taro corm starch.

The hydrothermal response of the three starches under excess-water conditions across the evaluated temperature range is shown in Figure 6. The gradual increase in temperature between 60 and 90 °C promoted granular expansion in all three starches, due to the interaction of the hydroxyl (-OH) groups of amylose and amylopectin with water molecules [39], as well as the hydration of the molecular chains during heating [50]. At 60 °C, no significant differences in SP were observed, suggesting still-limited gelatinization, with initial hydration and restricted water penetration into the amorphous regions of the granule. At 70 °C, MCS exhibited an increase in SP, which could indicate greater initial susceptibility to water penetration and granule expansion, possibly associated with a more advanced gelatinization process. In contrast, BPS exhibited a lower SP, likely related to its higher amylose content and larger granule size [122,123]. These are characteristics that may restrict water diffusion within the granule, favoring greater chain interaction, the formation of organized helical structures, and structural networks with compact bonds [51,124]. TCS also exhibited relatively low values at 70 °C, despite its lower amylose content. Although the type A crystalline pattern of TCS is primarily associated with a higher proportion of short amylopectin chains, its length distribution also includes fractions of long chains, which could promote a more stable molecular organization and, consequently, initially limit granular expansion [125]. At higher temperatures (80–90 °C), the three starches reached similar SP values, suggesting that, under these conditions, the effect of heating on granular disassembly predominated over the initial structural differences among the starches.

At 60 °C, MCS exhibited a lower WSI, which could be attributed to the influence of the lipids and proteins present (Table 1), which might form a surface barrier around the granules and restrict the release of soluble components. Consistent with this, several studies have reported an increase in WSI following the removal of minor fractions associated with starch [51]. On the other hand, BPS showed a decrease in WSI to 10.30% at 90 °C, a behavior similar to that observed in native cassava starch, whose WSI decreased from 43.5% to 33.5% [126], This reduction could indicate a limited additional release of soluble components, possibly associated with greater stability of the granular structure. In contrast, TCS exhibited a marked increase in WSI, reaching 33.99% at 90 °C, suggesting greater disorganization of the granular structure and increased release of soluble components as a result of the alteration of crystalline regions and the progressive breakdown of intermolecular interactions [127]. Furthermore, Refs. [44,128], note that this behavior could be related to its lower amylose content and granule size, characteristics that could contribute to greater susceptibility to structural disorganization during heating.

The increase in temperature promoted water absorption, associated with the progressive disorganization of the amylopectin double helices during heating [129]. At 70 °C, MCS exhibited the highest WAI, consistent with its high SP, while its low WSI suggests adequate water-retention capacity of the granular structure. BPS exhibited similar behavior, reaching its highest WAI at 90 °C and significantly low WSI values. In contrast, TCS showed a decrease in WAI at 90 °C, suggesting a greater loss of granule structural integrity [92] corresponding to the simultaneous increase in WSI.

### 3.8. Starch Pasting Behavior

The viscosity profiles show significantly different behavior (*p* < 0.05) among the starches studied, and these trends are summarized in Table 6. These differences result in variations in swelling capacity, stability during heating, and the degree of structural reorganization during cooling, enabling the integrated adoption of intrinsic rheological properties in the evaluation of performance and final applicability under processing conditions [42].

**Figure 7:**
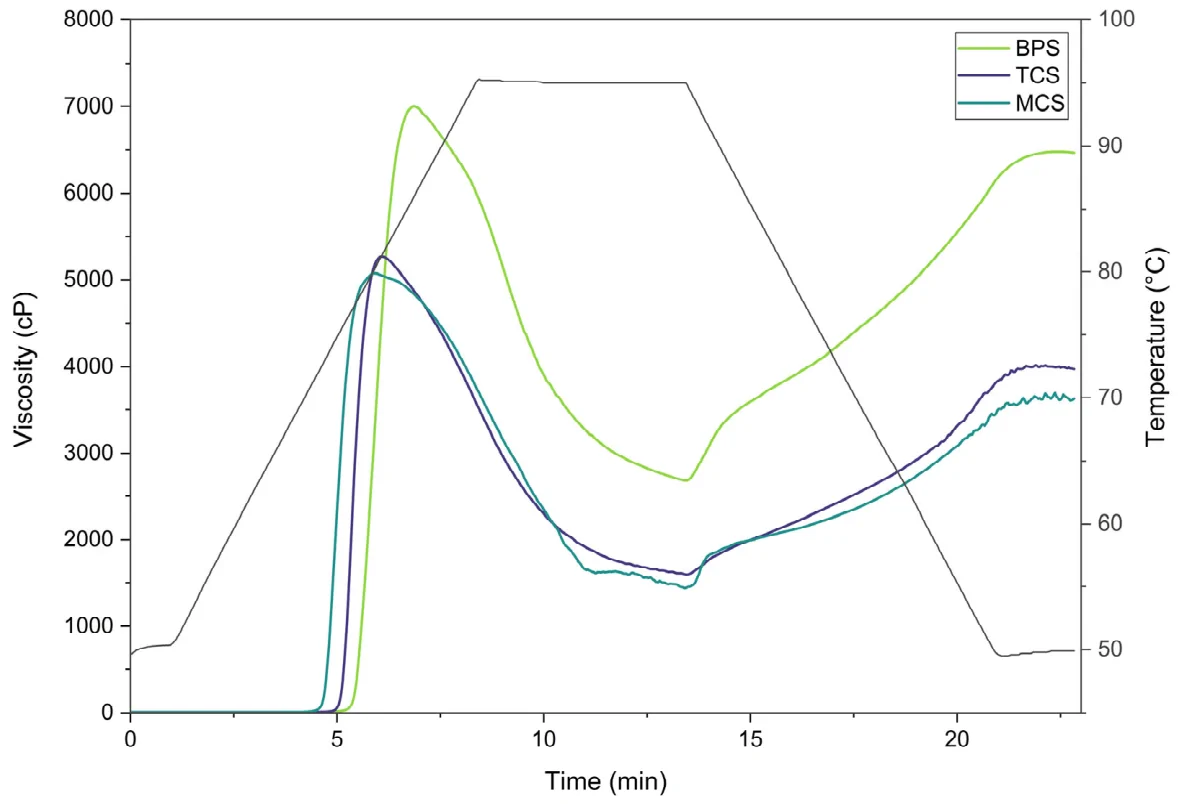
Viscosity curve during gelatinization of BPS: banana pulp starch; MCS: mango cotyledon starch; TCS: taro corm starch.

Figure 7 shows distinct viscosity profiles for the three starches, characterized by an initial increase associated with the hydration and expansion of the granules until a thermal plateau is reached [51], followed by a decrease during heating and a subsequent increase in viscosity during cooling, related to the reassociation of the polymer chains [80,130].

The quantitative parameters (Table 6) supported the differences observed in the viscosity profiles. The significant variations in PT indicate differences in the behavior of the granules during the gelatinization process [131]. The higher TCS value recorded (72.80 °C) suggests that these granules require a greater input of thermal energy to initiate gelatinization. On the other hand, BPS exhibited a significantly higher PV (7003.67 cP), reflecting a high capacity for granular expansion and viscosity development during heating, associated with the release of amylose into the surrounding liquid matrix [132]. This behavior could result from the balance between the intragranular forces that maintain the granule’s integrity during swelling and the intermolecular interactions that promote viscosity development, allowing high PV values to be reached before a significant loss of granular structure [42]. Consistently, the shift toward higher values for temperature (86.1 °C) and peak time (6.88 min) values in the BPS indicates that maximum viscosity development occurs under more demanding thermal conditions and with slower gelatinization kinetics. However, this behavior differs from that reported for starches from yellow cassava cultivars [133], where a high amylose content can restrict granule swelling, indicating that the described rheological response does not depend solely on the amount of amylose present but may also be modulated by factors such as granule size, the presence of associated components [134], and the structure and distribution of amylopectin chains [135]. In contrast, MCS exhibited the lowest PV value (5079.67 cP), accompanied by a lower gelatinization temperature (80.20 °C) and peak time (5.90 min), indicating earlier gelatinization and lower viscosity development during heating, possibly related to interactions between the starch and the lipid and protein content present [37].

The maximum viscosities (PV) of MCS, TCS, and BPS ranged from 5079 to 7003 cP. These values fall within the range reported for various conventional starches. For potato starches, values of 4350 to 6800 cP have been reported for varieties grown in India [136]. Similarly, values of 5174, 3380, and 6179 cP have been reported for plantain, rice, and potato starches, respectively [137]. However, the PV values obtained in this study were lower than those reported for native potatoes from Tureban Co., Ltd., Korea (8028 cP) [130], as well as for different potato varieties grown in Peru (14583–19450 cP) [120].

Similarly, for corn starches, PV values of 368, 18,300, and 13,300 cP have been reported for starches with high, waxy, and normal amylose content, respectively [138]. The wide variation observed even among starches from the same species highlights the possible influence of both their composition and the conditions used during the analysis. In particular, these authors observed a decrease in PV as amylose content increased and noted that the heating rate can also modify this parameter, with higher PV values recorded at lower heating rates [138]. However, this inverse relationship between amylose content and PV was not observed in the present study. Thus, the evidence reported in [58] indicates that the relationship between amylose content and PV is not always inverse, with some reports even showing opposite trends among groups of varieties; therefore, these differences among botanical sources may be related to the structural and physicochemical characteristics specific to each starch, which can alter the behavior of the granules during gelatinization.

BPS exhibited the highest BV (4325.33 cP), indicating a marked decrease in viscosity after reaching the peak and, therefore, greater susceptibility to granule disintegration under prolonged heating and agitation conditions. A similar pattern has been reported for potato and cassava starches, with a high degree of expansion but low resistance to thermal and mechanical stress [134]. In contrast, MCS exhibited a lower BV, indicating a reduced ability to form highly viscous structures, but also a lower susceptibility to collapse under severe conditions [42]. During cooling, BPS reached the highest values of FV (6467.67 cP) and SV (3789.67 cP), indicating a greater tendency toward polymer chain reassociation and the development of more rigid structures, a behavior that may be favored by the relatively linear nature of amylose [37,132]. This structural reorganization can contribute to undesirable textural changes in starch-rich products (bread, noodles, cakes, and cookies), such as loss of elasticity, hardening, coarseness, brittleness, and product staling [139]. In contrast, MCS and TCS exhibited significantly lower FV and SV values, suggesting a reduced capacity for molecular reassociation, which can be explained by their lower amylose content and granule size, factors that may have reduced the capacity for molecular reassociation during cooling. Likewise, the presence of non-starch traces may have exerted an inhibitory effect on starch retrogradation kinetics, due to the competition with water molecules, increased physical space between molecular chains, and steric hindrance [140,141]. This response has also been reported in commercial taro starch [142].

### 3.9. Dynamic Properties of Starch

**Figure 8:**
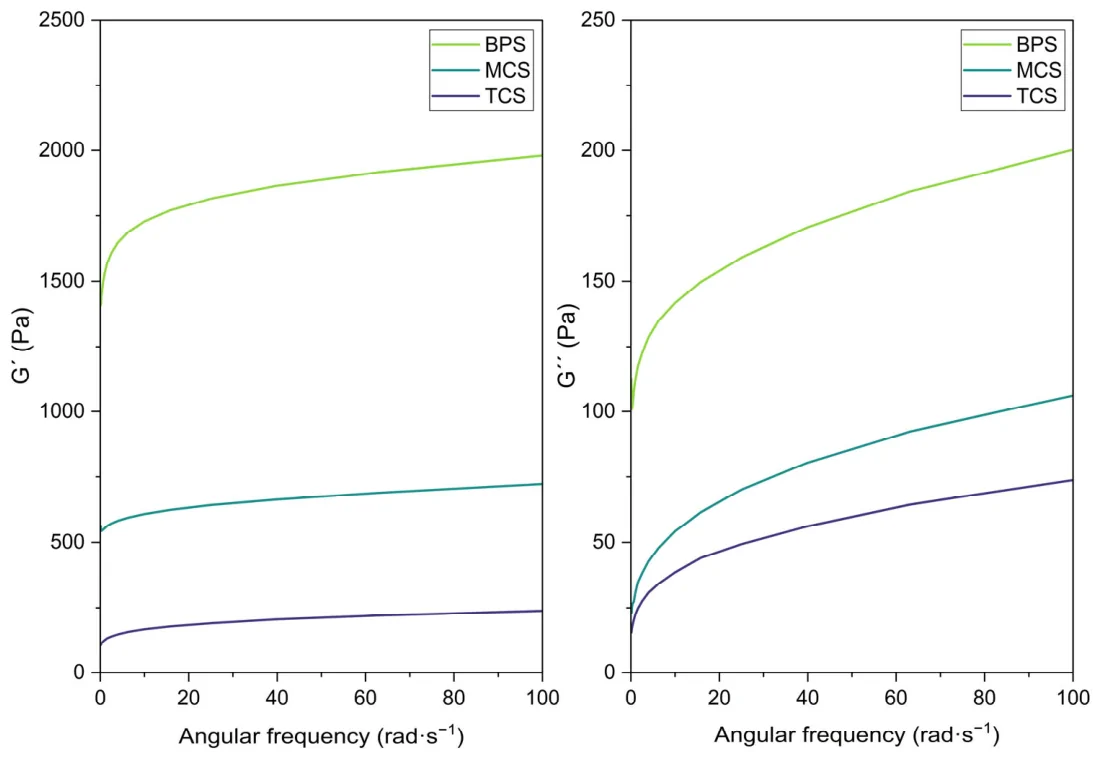
Dynamic response of storage modulus (G’) and loss modulus (G″) for BPS (banana pulp starch), MCS (mango cotyledon starch), and TCS (taro corm starch) gels.

The viscoelastic behavior of native starches, determined by angular frequency sweep of their gels, is summarized in Figure 8 and Table 7. The data obtained show significant variations among the samples across the entire frequency range analyzed, revealing differences in how each starch system responds to applied oscillatory deformations.

For all starch samples, the G’ and G″ moduli increased as the scanning frequency range increased, indicating a response dependent on the applied strain scale [135]. Within the evaluated frequency range, the samples maintained a consistent order of BPS > MCS > TCS for both moduli; this trend was preserved without any relative change in response, implying a sensitivity to the sample’s internal structure as well as to the angular oscillation velocity used, which is capable of determining the intensity of the interactions responsible for the elastic and viscous behavior of the starches [143].

BPS exhibited the highest values in the G’ = 1533.80 Pa and G” = 112.14 Pa modules, followed by MCS and TCS. This behavior suggests a continuous, solid-type network [144], characteristic of firmer gels with greater resistance to deformation [145,146]. The higher viscoelastic response of BPS may be related to its higher amylose content, which favors the interaction and cross-linking of the chains released during gelatinization, promoting the development of a more interconnected polymeric structure [147]. These associations promote greater structuring of the system, which is reflected in a greater capacity to store energy and, therefore, in an increase in G′ [148,149]. This interpretation is consistent with the greater setback observed for BPS (Table 6), which is associated with the reassociation of starch chains during cooling. Differences in granular and crystalline characteristics can modify water accessibility, the degree of swelling, and molecular reorganization during gelatinization [147,150,151]. In turn, the distribution of amylopectin chains can affect the organization of starch during cooling, since a higher proportion of long chains has been associated with higher viscoelastic moduli [147,148].

The tan δ analysis of the samples suggests that MCS (0.06) and BPS (0.07) exhibited solid behavior with a more stable structure [36]. In contrast, TCS, with a significantly higher value (0.18), revealed a greater viscous contribution. However, all samples met the conditions (G’ > G”) and (tan δ < 1), confirming the predominance of elastic behavior over viscous behavior, a characteristic of weak gel systems that are susceptible to progressive breakdown as deformation increases [152]. This is associated with the formation of a three-dimensional network consisting of leached amylose chains and swollen granules [153]. A characteristic widely described in starches from various botanical sources such as potato, corn, cassava, wheat, peas, beans, and green peas [150,154,155], unripe plantains [92], avocado seeds [156] and mango seeds [36]. These properties are advantageous for formulating foods with a smooth texture and stability during storage [156].

### 3.10. Calorimetric Analysis of Starch

**Figure 9:**
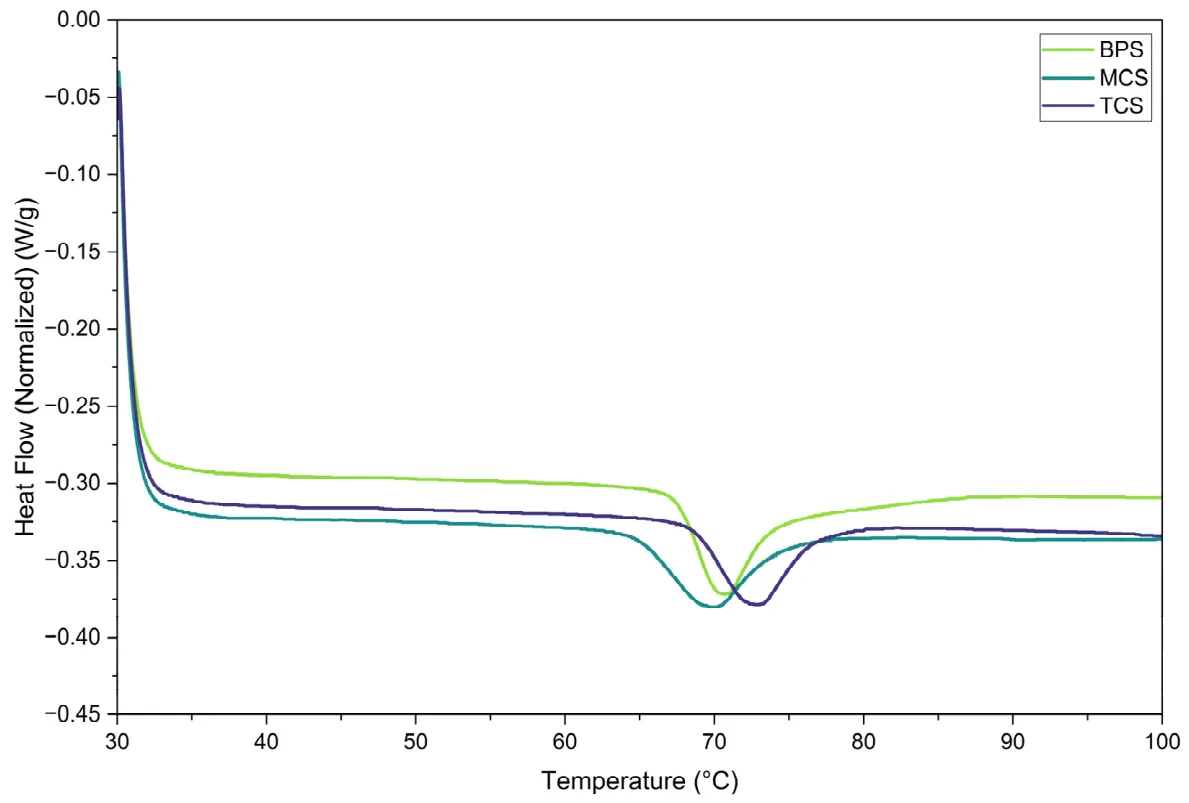
DSC thermograms of banana pulp starch (BPS); mango cotyledon starch (MCS) and taro corm starch (TCS).

The thermal profiles obtained by differential scanning calorimetry (DSC) presented in Table 8 revealed an endothermic transition associated with starch gelatinization (Figure 9), a process in which the crystalline regions of the granule are disrupted by the breaking of hydrogen bonds and the destabilization of the amylopectin double helices [157]. The thermal transition parameters showed significant differences (*p* < 0.05) associated with the origin and intrinsic characteristics of each botanical species.

The DSC thermograms shown in Figure 9 corresponded to an irreversible structural “order-to-disorder” transition in starch induced by the simultaneous action of heat and water, a process associated with the gradual entry of water molecules into the granule and the weakening of the intermolecular interactions responsible for maintaining the crystalline order [158].

According to the thermal parameters described in Table 8, TCS exhibited the highest values for *T*_o_ (68.48 °C), *T*_p_ (72.86 °C), and *T*_c_ (77.29 °C), indicating that its ordered structures require a greater amount of thermal energy to initiate the melting of the weaker crystalline zones, carry out the main melting of the crystalline population, and melt the more stable crystalline structures of the starch during the gelatinization process [159]. This could be associated with greater stability of the amylopectin double helices in the starch, consistent with its reported higher crystallinity. Likewise, Refs. [92,160] note that there is a direct relationship between the gelatinization temperature and the length of the amylopectin side chains. In contrast, MCS exhibited the lowest values for *T*_o_ (64.79 °C) and *T*_p_ (70.03 °C), indicating lower thermal stability of the crystalline regions and earlier gelatinization, which is consistent with the results presented in Table 4 and Table 6. This behavior could be related to the presence of non-starch components [161]. It has been reported that lipids, by forming complexes with amylose, reduce the energy required during gelatinization, which explains the low ∆*H*_gel_ (3.79 Jg^−1^) observed, in contrast to BPS; likewise, proteins can influence the interactions between water and starch granules, affecting the thermal behavior of the starch [162]. Furthermore, the study reported in [163] suggests that a higher proportion of short amylopectin chains results in less stable crystalline structures and lower ∆*H*_gel_ values. However, the intermediate transition temperature (*T*_c_ = 75.29 °C) observed indicates that a fraction of the crystalline structure remained stable up to relatively high temperatures. Meanwhile, the intermediate values of *T*_o_ (67.45 °C) and *T*_p_ (70.76 °C) for BPS indicate moderate thermal stability of the crystalline regions. However, the lower *T*_c_ (74.09 °C) observed suggests that the gelatinization process concluded within a narrower temperature range, which could be related to its high amylose content. As discussed in the study reported in [114], which describes an increasing order of the gelatinization range (*T*_c_–*T*_o_) as the amylose content decreases.

The high ∆*H*_gel_ value (5.56 Jg^−1^) for BPS is consistent with the findings reported in [114], for Brazilian potato starches, where high ∆*H*_gel_ values were not necessarily associated with higher RC. Therefore, this behavior could depend on additional structural factors such as the quantity, length, and interaction of amylopectin chains, as well as the proportion and shape of the granules [114,164]. In contrast, the lower ∆*H*_gel_ value observed in TCS (3.91 Jg^−1^) could be related to the size of its granules, given that a smaller granular size may favor molecular disorganization at lower energy requirements, due to a reduction in the effective length of the amylopectin double helices [165]. The values obtained are comparable to those reported in [120] for native Peruvian potato starches (∆*H*_gel_ = 3.60–6.62 Jg^−1^). As discussed in [42] in terms of biopolymer applications, low ∆*H*_gel_ values are advantageous for industrial applications, as they are associated with lower energy requirements during thermal processing.

The gelatinization temperatures of BPS were higher than those reported by [92] with results of *T*_o_ = 57.33–62.51 °C; *T*_p_ = 60.64–65.17 °C; *T*_c_ = 66.02–69.74 °C. Meanwhile, ∆*H*_gel_ was lower than that of green plantain starches (8.04–9.47 J g^−1^) from Colombian varieties [166] and (11.18-13.85 Jg^−1^) from Tanzanian varieties [92]. Likewise, starches from ripe mango seeds of Colombian cultivars exhibited higher peak temperatures (83.83 and 84.75 °C) and lower enthalpies (1.56 and 1.66 Jg^−1^) [167] than those reported by MCS, in contrast to the findings reported in [168], which showed higher DSC parameters (*T*_o_ = 69.89 °C; *T*_p_ = 75.89 °C; *T*_c_ = 86.30 °C; ∆*H*_gel_ = 9.04 Jg^−1^) in starches from green mango seeds from Mexico. Meanwhile, native taro starches from China exhibited higher thermal values (*T*_o_ = 77.33 °C; *T*_p_ = 81.17 °C; *T*_c_ = 85.01 °C; ∆*H*_gel_ = 12.83 Jg^−1^) than those described in TCS, whereas the study reported in [169], obtained low values for *T*_o_ (52.09 °C) and *T*_p_ (61.29 °C) and high values for *T*_c_ (82.80 °C) and ∆*H*_gel_ (14.58 Jg^−1^) in starches extracted from Indian taro varieties. Similarly, the study reported in [170] evaluated different varieties of native potatoes, and differences were observed in the temperatures (*T*_o_ = 54.1–56.1 °C; *T*_p_ = 61.8–66.4 °C; *T*_c_ = 73–79.1 °C), along with differences in the enthalpies of gelatinization (∆*H*_gel_ = 6.84 Jg^−1^). This reaffirms the influence of structural factors such as the distribution and organization of the amorphous and crystalline regions of starch and the strength of hydrogen bonds as well as factors associated with environmental conditions, variety, and physiological state, on DSC thermal profiles.

### 3.11. Thermogravimetric Analysis of Starch

**Figure 10:**
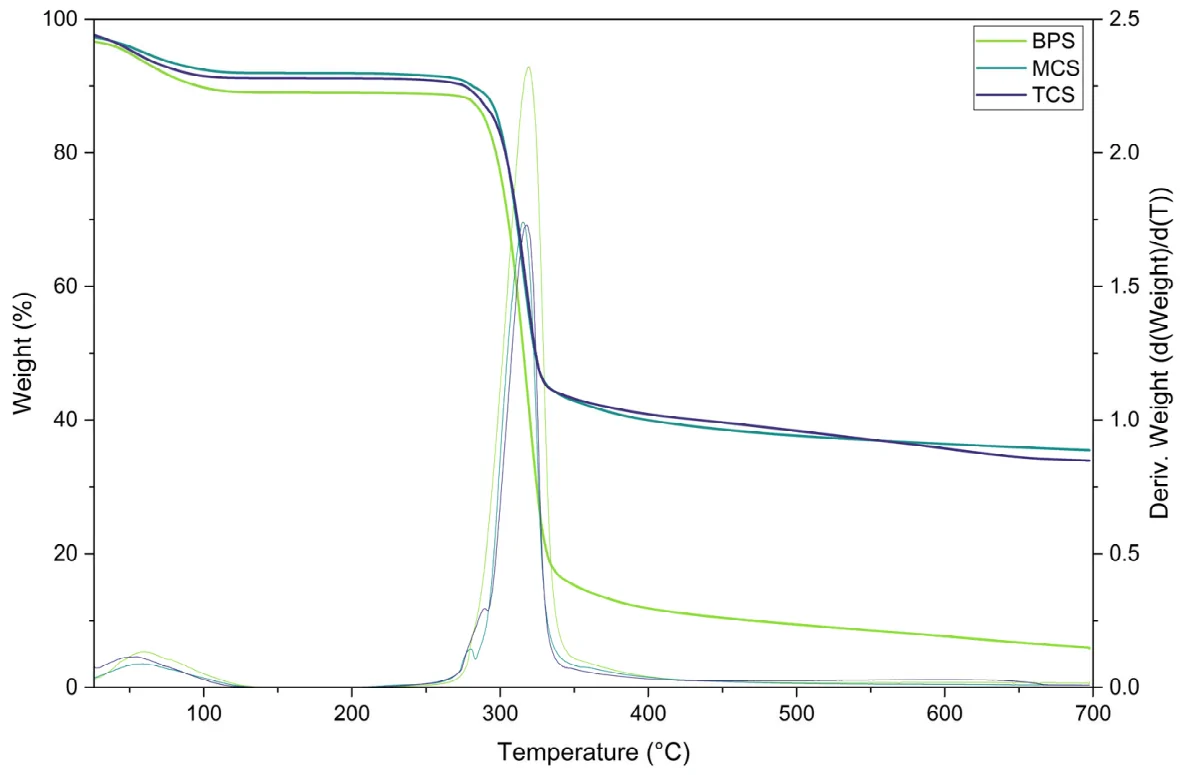
Curves TG/DTG of BPS: banana pulp starch; MCS: mango cotyledon starch; TCS: taro corm starch.

The behavior and heat resistance of the starches, as defined by the thermograms and derived kinetic parameters described in Figure 10 and Table 9, allowed for the identification of two main thermal decomposition events dominated by an inert N_2_ atmosphere, reflecting divergent physical and chemical transitions (*p* < 0.05) between the polysaccharide samples within the programmed temperature range.

Thermogravimetric profiles show that the TG/DTG curves maintain a plateau of relative stability from 150 °C to 250 °C, interrupted by an initial dip due to water loss, linked to the evaporation of free surface water and the breaking of weak hydrogen bonds, which allow the release of water structurally bound to the hydrophilic groups of the molecular chains as the temperature rises [109], and by a zone of maximum pyrolytic activity, associated with the chemical destruction of starch through the breaking of glycosidic bonds and the fragmentation of pyranose rings, which generate the combined volatilization of gaseous compounds (CO_2_, CO, and H_2_O) and low-molecular-weight organic compounds (aldehydes, alcohols, carboxylic acids, ketones, and furan derivatives), in addition to the formation of carbonaceous material [171,172]. The curve corresponding to BPS exhibits a more pronounced drop in the region of maximum thermal degradation, suggesting a more accelerated depolymerization process. At temperatures above 500 °C, the DTG curves showed a mass loss rate close to zero, confirming that the process of maximum starch degradation had concluded. However, the TG curves did not reach a plateau of absolute constant mass, still exhibiting a slight negative slope up to 700 °C, associated with gradual carbonization kinetics accompanied by residual degassing [171].

The first thermal event, occurring in the temperature ranges of 25.64 to 134.65 °C, 25.56 to 130.77 °C, and 24.77 to 117.50 °C for BPS, MCS, and TCS, respectively, is attributed to the desorption of water from the polymer matrix [173]. Although slight variations were observed in the degradation temperatures of the event (*T*_deg1_) and in mass loss (Δ*m*), these differences were not statistically significant (*p* > 0.05), indicating similar thermal behavior during the initial stage of water loss among the samples, which could be related to their similar moisture content (Table 1) [172]. The study reported in [114] indicates that water sorption capacity depends on the crystalline pattern and degree of crystallinity, as well as on the amylopectin content, since a type B pattern typically retains a greater amount of water within its structure [103], whereas the degree of crystallinity has an inverse relationship with the availability of free OH groups, and amylopectin branching promotes hydrogen bonding with water molecules [174]. Therefore, the similarity observed in the mass loss during the first event suggests a comparable amount of adsorbed water and a similar (water–starch) interaction among the samples, rather than differences in structure inherent to the botanical source.

The temperature ranges of 254.20 to 397.15 °C, 252.25 to 397.07 °C, and 244.46 to 397. 12 °C for BPS, MCS, and TCS associated with the second thermal event represented the maximum mass loss due to molecular breakdown of starch and organic compounds [158]. Although BPS exhibited lower crystallinity and lower FTIR band intensity, it reached the highest maximum degradation temperature (*T*_deg2_) at 319.27 °C, indicating greater macromolecular stability and resistance during heating [175], suggesting that the reported thermal stability may have depended primarily on factors such as higher amylose content and particle size, due to the ability to form more stable intermolecular associations and double-helix structures [176], thereby increasing the energy required for thermal degradation of the polymer matrix. Likewise, the lower ash and protein content promoted more complete degradation and more efficient organic volatilization (Δ*m* = 86.52%) during heating, which explains the significantly low final residue (5.98%) reported.

In contrast, TCS exhibited a *T*_deg2_ of 317.66°4C, indicating a higher activation energy required for the breakdown of the starch structure, which is statistically comparable to BPS; this can be attributed to its lower amylose content, as well as its higher relative crystallinity. It has been reported that a higher amylopectin content, due to the high branching and density of its chains, requires greater thermal energy to initiate significant structural breakdown; furthermore, a higher amorphous fraction can reduce thermal stability [114]. On the other hand, the lower mass loss (54.84%) and high final residue (33.93%) recorded for TCS can be explained by the higher content of associated inorganic components (ash) [120]. For its part, MCS recorded the lowest degradation temperature of 315.58 °C, with mass loss (56.13%) and final residue (35.54%) parameters that were not significant compared to TCS, indicating pyrolysis mechanisms slowed by chemical and structural constraints. According to [173], systems with lower starch content exhibit higher levels of mineral traces after excessive heat treatments.

The maximum degradation temperatures of the starches (*T*_deg2_ = 315–319 °C) were lower than those reported for conventional corn starches (335.1 °C) [177] and sweet potato starches (331.6–344.7 °C) [157,177] indicating lower thermal resistance compared to these starchy sources. However, the temperatures obtained were higher than those reported for different Andean potato cultivars native to Colombia (296–303.9 °C) [173], demonstrating relatively high thermal stability. These differences can be attributed to variations in their botanical origin and in the molecular structure of the starches, such as the amylose:amylopectin ratio, the relative degree of crystallinity, and the organization of the polymer chains. Although the Tdeg_2_ values obtained were lower than those observed in some physically modified potato starches, the recorded values indicate a structure with moderate thermal stability; thus, the lower degradation temperature compared to modified starches could be related to a lower degree of reorganization and intermolecular interaction [157].

## 4. Conclusions

The results revealed distinct profiles among the evaluated starches in terms of composition, structure, and functional behavior. The low moisture and ash contents reflected a favorable physicochemical composition, while differences in protein and lipid content revealed characteristics specific to each source and the extraction process. TCS stood out for its smaller particle size and greater structural organization. MCS and TCS exhibited type A crystallinity, while BPS showed a type C pattern and lower crystalline order, consistent with FTIR results. The BPS stood out for its higher Δ*H*_gel_ and *T*_deg2_ values, which are associated with a greater energy requirement for gelatinization and greater resistance to thermal degradation. However, its high PV, breakdown, and retrogradation values indicated that it was more susceptible to the disintegration of swollen granules and to chain reassociation during cooling. These characteristics support its potential as a thickener and structuring agent, although their high retrogradation could limit applications requiring prolonged textural stability, such as in baked goods. MCS exhibited the lowest values for WSI, PV, retrogradation, Δ*H*_gel_, and *T*_deg2_, along with lower swelling capacity and viscosity development. This behavior suggests lower energy requirements for gelatinization and lower thermal resistance, as well as limited chain reassociation, characteristics that could be advantageous in formulations requiring moderate viscosity and low retrogradation. TCS exhibited higher WSI, lower WAI, high crystallinity, higher *T*_o_ and *T*_p_, and a high Tdeg_2_ comparable to BPS, demonstrating greater resistance to the onset of gelatinization and to thermal degradation, although with greater granular disruption during heating. Its low retrogradation and smaller particle size could favor formulations with limited reassociation and a homogeneous appearance.

Overall, the differences observed among the three starches reveal distinct functional profiles that can be leveraged according to specific processing and application requirements. These results support the potential of these unconventional starch sources and contribute to expanding knowledge about Peruvian raw materials for the development of ingredients and biopolymer materials.

## Figures and Tables

**Table 1:** Chemical composition and amylose fraction of native starches.

Starch	Moisture (%)	Ash (%)	Proteins (%)	Lipids (%)	Carbohydrates (%)	Amylose (%)
BPS	7.90 ± 0.30 ^a^	0.10 ± 0.00 ^b^	1.13 ± 0.04 ^c^	0.11 ± 0.05 ^b^	90.75 ± 0.32 ^a^	25.92 ± 0.48 ^a^
MCS	7.51 ± 0.11 ^ab^	0.12 ± 0.01 ^b^	1.93 ± 0.04 ^a^	1.10 ± 0.05 ^a^	89.35 ± 0.18 ^b^	21.97 ± 1.60 ^a^
TCS	7.30 ± 0.18 ^b^	0.28 ± 0.01 ^a^	1.69 ± 0.03 ^b^	0.10 ± 0.01 ^b^	90.63 ± 0.19 ^a^	16.83 ± 0.28 ^b^

BPS: banana pulp starch; MCS: mango cotyledon starch; TCS: taro corm starch. Data set expressed as mean ± calculated dispersion. Differentiated alphabetical notations reveal (*p* < 0.05).

**Table 2:** CIELab coordinates and colorimetric sub-indices of native starches.

Starch	L*	a*	b*	WI	YI	C*	h°
BPS	84.53 ± 0.23 ^c^	4.10 ± 0.02 ^a^	12.40 ± 0.35 ^a^	79.75 ± 0.11 ^c^	20.96 ± 0.54 ^a^	13.06 ± 0.34 ^a^	71.69 ± 0.41 ^c^
MCS	90.04 ± 0.28 ^b^	1.47 ± 0.06 ^b^	11.72 ± 0.08 ^b^	84.54 ± 0.19 ^b^	18.60 ± 0.14 ^b^	11.82 ± 0.09 ^b^	82.85 ± 0.27 ^b^
TCS	98.30 ± 0.08 ^a^	−0.07 ± 0.03 ^c^	2.99 ± 0.08 ^c^	96.56 ± 0.03 ^a^	4.35 ± 0.11 ^c^	2.99 ± 0.08 ^c^	91.40 ± 0.60 ^a^

L*: Lightness; a*: red-green coordinate; b*: yellow-blue coordinate; WI: whiteness index; YI: yellowness index; C*: Chroma; h°: hue angle. BPS: banana pulp starch; MCS: mango cotyledon starch; TCS: taro corm starch. Data set expressed as mean ± calculated dispersion. Differentiated alphabetical notations indicate significance (*p* < 0.05).

**Table 3:** Particle size metrics of native starches.

Starch	D50 (µm)	D90 (µm)	D[4,3] (µm)	D[3,2] (µm)	Span
BPS	22.40 ± 1.16 ^a^	41.50 ± 1.88 ^a^	24.37 ± 0.93 ^a^	4.50 ± 3.70 ^a^	1.63 ± 0.41 ^a^
MCS	13.48 ± 0.31 ^b^	28.11 ± 0.66 ^b^	15.21 ± 0.25 ^b^	1.34 ± 0.42 ^a^	2.06 ± 0.04 ^a^
TCS	4.43 ± 0.05 ^c^	8.01 ± 0.31 ^c^	6.64 ± 0.55 ^c^	1.51 ± 0.10 ^a^	1.69 ± 0.10 ^a^

D50 and D90: particle diameters below which 50% and 90% of the cumulative particle volume is distributed, respectively; D[4,3] and D[3,2]: volume-weighted and surface-area-weighted mean diameters; Span: width of the particle size distribution; BPS: banana pulp starch; MCS: mango cotyledon starch; TCS: taro corm starch. Data set expressed as mean ± calculated dispersion. Differentiated alphabetical notations reveal (*p* < 0.05).

**Table 4:** Crystalline organization parameters of native starches.

Starch	Main Peaks 2θ (°)	Relative Crystallinity (%)
BPS	5.78; 15.28; 17.2; 23.18	29.67 ± 0.29 ^c^
MCS	15.2; 17.22; 18.12; 23.16	31.72 ± 0.31 ^b^
TCS	15.08; 16.98; 17.94; 22.96	36.68 ± 0.26 ^a^

BPS: banana pulp starch; MCS: mango cotyledon starch; TCS: taro corm starch. Data set expressed as mean ± calculated dispersion. Differentiated alphabetical notations reveal (*p* < 0.05).

**Table 5:** Starch behavior in water of native starches.

Starch	Temperature [°C]	SP (g/g)	WSI (%)	WAI (g/g)
BPS	60	2.34 ± 0.14 ^e^	4.21 ± 0.76 ^f^	2.24 ± 0.14 ^f^
	70	6.48 ± 0.08 ^d^	9.41 ± 0.78 ^d^	5.87 ± 0.02 ^e^
	80	14.17 ± 1.12 ^ab^	17.03 ± 1.72 ^bc^	11.76 ± 0.98 ^b^
	90	16.35 ± 0.18 ^ab^	10.30 ± 0.43 ^d^	14.66 ± 0.23 ^a^
MCS	60	2.45 ± 0.31 ^e^	0.68 ± 0.24 ^g^	2.43 ± 0.31 ^f^
	70	9.74 ± 0.51 ^c^	8.89 ± 0.48 ^de^	8.87 ± 0.42 ^cd^
	80	13.64 ± 0.30 ^b^	10.74 ± 1.44 ^d^	12.18 ± 0.19 ^b^
	90	16.42 ± 1.66 ^ab^	18.69 ± 0.22 ^b^	13.35 ± 1.33 ^ab^
TCS	60	3.02 ± 0.06 ^e^	5.011 ± 0.19 ^f^	2.87 ± 0.06 ^f^
	70	7.00 ± 0.43 ^cd^	5.66 ± 0.23 ^ef^	6.61 ± 0.41 ^de^
	80	14.47 ± 2.45 ^ab^	14.09 ± 0.49 ^c^	12.44 ± 2.17 ^ab^
	90	16.58 ± 0.22 ^a^	33.99 ± 2.90 ^a^	10.95 ± 0.62 ^bc^

SP: swelling power; WSI: water solubility index; WAI: water absorption index; BPS: banana pulp starch; MCS: mango cotyledon starch; TCS: taro corm starch. Data set expressed as mean ± calculated dispersion. Differentiated alphabetical notations reveal (*p* < 0.05).

**Table 6:** Evolution of native starch viscosity.

Starch	PT (°C)	PV (cP)	Temperature at PV (°C)	Peak Time (min)	TV (cP)	BV (cP)	FV (cP)	SV (cP)
BPS	72.33 ± 0.21 ^b^	7003.67 ± 19.09 ^a^	86.10 ± 0.17 ^a^	6.88 ± 0.02 ^a^	2678.00 ± 59.91 ^a^	4325.33 ± 46.29 ^a^	6467.67 ± 25.97 ^a^	3789.67 ± 34.44 ^a^
MCS	70.03 ± 0.15 ^c^	5079.67 ± 13.58 ^c^	80.20 ± 0.20 ^c^	5.90 ± 0.03 ^c^	1426.00 ± 80.90 ^c^	3653.33 ± 69.62 ^b^	3630.00 ± 69.66 ^c^	2204.00 ± 47.62 ^c^
TCS	72.80 ± 0.10 ^a^	5265.33 ± 15.57 ^b^	81.33 ± 0.12 ^b^	6.09 ± 0.02 ^b^	1594.67 ± 7.64 ^b^	3671.67 ± 12.74 ^b^	3972.33 ± 66.38 ^b^	2377.67 ± 58.79 ^b^

PT: Pasting Temperature; PV: Peak Viscosity; TV: Trough viscosity; BV: Breakdown viscosity (PV-TV); FV: Final Viscosity; SV: Setback Viscosity (FV-TV). BPS: banana pulp starch; MCS: mango cotyledon starch; TCS: taro corm starch. Data set expressed as mean ± calculated dispersion. Differentiated alphabetical notations reveal (*p* < 0.05).

**Table 7:** Viscoelastic parameters of native starch.

Starch	G’ (Pa)	G″ (Pa)	Tan δ
BPS	1533.80 ± 247.48 ^a^	112.14 ± 7.08 ^a^	0.07 ± 0.01 ^b^
MCS	556.15 ± 40.90 ^b^	30.64 ± 1.43 ^b^	0.06 ± 0.00 ^b^
TCS	126.92 ± 14.27 ^c^	22.45 ± 0.52 ^b^	0.18 ± 0.02 ^a^

Results expressed at an angular frequency of 1 rad·s^−1^. G′: Storage modulus; G″: Loss modulus; tan δ: Loss tangent calculated from the ratio (G″/G′). BPS: banana pulp starch; MCS: mango cotyledon starch; TCS: taro corm starch. Data set expressed as mean ± calculated standard deviation. Differentiated alphabetical notations indicate (*p* < 0.05).

**Table 8:** DSC thermal transition parameters of native starches.

Starch	*T*_o_ (°C)	*T*_p_ (°C)	*T*_c_ (°C)	∆*H_g_*_el_ (Jg^−1^)
BPS	67.45 ± 0.11 ^b^	70.76 ± 0.28 ^b^	74.09 ± 0.45 ^c^	5.56 ± 0.32 ^a^
MCS	64.79 ± 0.13 ^c^	70.03 ± 0.10 ^c^	75.29 ± 0.10 ^b^	3.79 ± 0.75 ^b^
TCS	68.48 ± 0.06 ^a^	72.86 ± 0.16 ^a^	77.29 ± 0.24 ^a^	3.91 ± 0.40 ^b^

*T*_o_: Onset Temperature; *T*_p_: Peak Temperature; *T*_c_: Conclusion Temperature; ∆*H*_gel_: Enthalpy of gelatinization; BPS: banana pulp starch; MCS: mango cotyledon starch; TCS: taro corm starch. Data set expressed as mean ± calculated dispersion. Differentiated alphabetical notations reveal (*p* < 0.05).

**Table 9:** Thermal decomposition events of native starches.

Starch	First Thermal Event			Second Thermal Event			Residual Weight (%)
	*T* _deg1_ (°C)	Intervale *T*_o_—*T*_f_ (°C)	Δm (%)	*T* _deg2_ (°C)	Intervale *T*_o_—*T*_f_ (°C)	Δ*m* (%)	
BPS	60.32 ± 3.20 ^a^	25.64–134.65	7.81 ± 1.28 ^a^	319.27 ± 0.36 ^a^	254.20–397.15	86.52 ± 1.51 ^a^	5.98 ± 0.58 ^b^
MCS	58.45 ± 7.94 ^a^	25.56–130.77	5.45 ± 1.89 ^a^	315.58 ± 0.53 ^b^	252.25–397.07	56.13 ± 0.15 ^b^	35.54 ± 0.95 ^a^
TCS	52.53 ± 9.24 ^a^	24.77–117.50	6.59 ± 2.08 ^a^	317.66 ± 1.24 ^a^	244.46–397.12	54.84 ± 0.93 ^b^	33.93 ± 4.16 ^a^

*T* _deg1_ and *T* _deg2_: First and second thermal degradation; Δ*m*: mass variation with respect to *T*_o_ and *T*_f_ of the thermal event; *T*_o_—*T*_f_: Start and end of the degradation curve; BPS: banana pulp starch; MCS: mango cotyledon starch; TCS: taro corm starch. Data set expressed as mean ± calculated dispersion. Differentiated alphabetical notations reveal (*p* < 0.05).

## Data Availability

The data presented in this study are available on request from the corresponding authors.

## Abbreviations

The following abbreviations are used in this manuscript:

AOAC	Association of Official Analytical Chemists
ATR	Attenuated Total Reflectance
CIELab	Commission Internationale de l’Éclairage L*a*b* color space
DSC	Differential Scanning Calorimetry
DTG	Derivative Thermogravimetry
FTIR	Fourier-Transform Infrared Spectroscopy
IA	Amylose Affinity Index
PSA	Particle Size Analysis
RC	Relative Crystallinity
SED	Secondary Electron Detector
SEM	Scanning Electron Microscopy
SP	Swelling Power
TG	Thermogravimetric Analysis
WAI	Water Absorption Index
WSI	Water Solubility Index
XRD	X-ray Diffraction
